# Electrostatic sheathing of lipoprotein lipase is essential for its movement across capillary endothelial cells

**DOI:** 10.1172/JCI157500

**Published:** 2022-03-01

**Authors:** Wenxin Song, Anne P. Beigneux, Anne-Marie L. Winther, Kristian K. Kristensen, Anne L. Grønnemose, Ye Yang, Yiping Tu, Priscilla Munguia, Jazmin Morales, Hyesoo Jung, Pieter J. de Jong, Cris J. Jung, Kazuya Miyashita, Takao Kimura, Katsuyuki Nakajima, Masami Murakami, Gabriel Birrane, Haibo Jiang, Peter Tontonoz, Michael Ploug, Loren G. Fong, Stephen G. Young

**Affiliations:** 1 Department of Medicine, David Geffen School of Medicine, UCLA, Los Angeles, California, USA.; 2 Finsen Laboratory, Rigshospitalet, Copenhagen, Denmark.; 3 Biotech Research and Innovation Centre (BRIC), University of Copenhagen, Copenhagen, Denmark.; 4 Children’s Hospital Oakland Research Institute, Oakland, California, USA.; 5 Department of Clinical Laboratory Medicine, Gunma University, Graduate School of Medicine, Maebashi, Gunma, Japan.; 6 Immuno-Biological Laboratories (IBL), Fujioka, Gunma, Japan.; 7 Division of Experimental Medicine, Beth Israel Deaconess Medical Center, Boston, Massachusetts, USA.; 8 Department of Chemistry, The University of Hong Kong, Hong Kong.; 9 Department of Pathology and Laboratory Medicine, UCLA, Los Angeles, California, USA.; 10 Department of Human Genetics, David Geffen School of Medicine, UCLA, Los Angeles, California, USA.

**Keywords:** Metabolism, Lipoproteins

## Abstract

GPIHBP1, an endothelial cell (EC) protein, captures lipoprotein lipase (LPL) within the interstitial spaces (where it is secreted by myocytes and adipocytes) and transports it across ECs to its site of action in the capillary lumen. GPIHBP1’s 3-fingered LU domain is required for LPL binding, but the function of its acidic domain (AD) has remained unclear. We created mutant mice lacking the AD and found severe hypertriglyceridemia. As expected, the mutant GPIHBP1 retained the capacity to bind LPL. Unexpectedly, however, most of the GPIHBP1 and LPL in the mutant mice was located on the abluminal surface of ECs (explaining the hypertriglyceridemia). The GPIHBP1-bound LPL was trapped on the abluminal surface of ECs by electrostatic interactions between the large basic patch on the surface of LPL and negatively charged heparan sulfate proteoglycans (HSPGs) on the surface of ECs. GPIHBP1 trafficking across ECs in the mutant mice was normalized by disrupting LPL-HSPG electrostatic interactions with either heparin or an AD peptide. Thus, GPIHBP1’s AD plays a crucial function in plasma triglyceride metabolism; it sheathes LPL’s basic patch on the abluminal surface of ECs, thereby preventing LPL-HSPG interactions and freeing GPIHBP1-LPL complexes to move across ECs to the capillary lumen.

## Graphical abstract

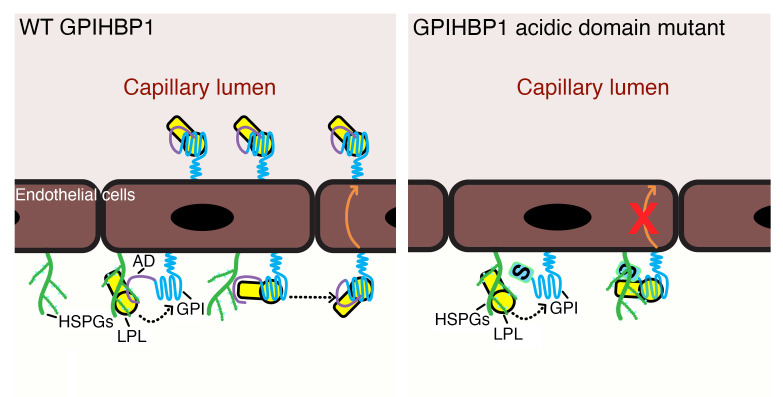

## Introduction

Glycosylphosphatidylinositol-anchored high density lipoprotein–binding protein 1 (GPIHBP1), which is expressed by capillary endothelial cells (ECs), is required for the intravascular processing of triglyceride-rich (TG-rich) lipoproteins (TRLs) by lipoprotein lipase (LPL) (1, 2). LPL is synthesized by adipocytes and myocytes and secreted into the interstitial spaces (3), but it functions to hydrolyze TGs along the luminal surface of capillary ECs. A key physiologic function of GPIHBP1 is to capture LPL from the subendothelial spaces and shuttle it across ECs to its site of action in the capillary lumen (2). In GPIHBP1-deficient (*Gpihbp1*^–/–^) mice, LPL never reaches the capillary lumen, resulting in impaired TRL processing and severe hypertriglyceridemia (chylomicronemia; refs. 2, 4).

The capture of interstitial LPL by GPIHBP1 on ECs is efficient, evident by the fact that most of the LPL in tissues of wild-type (WT) mice is located on capillary ECs (2). In *Gpihbp1*-deficient mice, LPL remains within the interstitial spaces, trapped there by electrostatic interactions between a large (~2400 Å^2^) basic patch on the surface of LPL (5) and negatively charged cell-surface heparan sulfate proteoglycans (HSPGs) (2, 6). The binding of LPL to interstitial HSPGs in *Gpihbp1*^–/–^ mice prevents the escape of catalytically active LPL into the systemic circulation (1, 2). Of note, the LPL-HSPG electrostatic interactions are transient (7), explaining why the LPL in WT mice is able to detach from HSPGs and move to more stable interactions with GPIHBP1 on ECs (6, 8).

GPIHBP1 is a member of the LU (Ly6/uPAR) protein superfamily. Like all LU family members, GPIHBP1 contains a 3-fingered cysteine-rich fold (LU domain) (9), but it is unique in having an amino-terminal intrinsically disordered acidic domain (AD) (1, 8, 10). The AD contains a long stretch of acidic residues (DEEDEDEVEEEE in human; DDDDDEEEEEE in mouse) and an adjacent tyrosine, which was shown to be sulfated in human GPIHBP1 (8).

GPIHBP1’s LU domain is required for LPL binding and trafficking across ECs (11, 12). Mutagenesis studies identified multiple LU domain residues required for LPL binding (12, 13). Also, autoantibodies against the LU domain were identified as a cause of some cases of acquired chylomicronemia (8, 14, 15). The in vivo relevance of the LU domain was firmly established by finding absent intravascular LPL and severe chylomicronemia in knockin mice harboring a single cysteine substitution in the LU domain (16, 17). A crystal structure of the GPIHBP1-LPL complex revealed that GPIHBP1’s LU domain binds, by hydrophobic contacts, to LPL’s carboxyl-terminal domain (5). *LPL* and *GPIHBP1* mutations that interfere with LPL-LU domain interactions prevent LPL transport and cause chylomicronemia (5, 10, 12, 18).

GPIHBP1’s disordered AD was not visualized in the crystal structure but was positioned to project across and interact with LPL’s basic patch (5). The basic patch is distant from both LPL’s catalytic pocket and the hydrophobic surface that interfaces with GPIHBP1’s LU domain (5). GPIHBP1’s AD is not required for LPL binding; a mutant human GPIHBP1 lacking the AD forms stable interactions with LPL (8, 19). However, biophysical studies with purified proteins raised the possibility that the AD could be important for TG metabolism (10, 20). First, surface plasmon resonance (SPR) studies revealed that the AD of human GPIHBP1 accelerates interactions between GPIHBP1 and LPL despite having little or no effect on the stability of the binding (i.e., negligible effects on the off-rate; ref. 19). Second, hydrogen-deuterium exchange/mass spectrometry (HDX-MS) studies revealed that GPIHBP1’s AD stabilizes LPL catalytic activity by limiting unfolding of LPL’s hydrolase domain (19, 21).

While the SPR and HDX-MS studies suggested that the AD could be important for TG metabolism in vivo, this idea has never been tested to the best of our knowledge. Furthermore, it has been difficult to make predictions about the functional relevance of the AD. For example, it seemed quite possible that the ability of GPIHBP1’s LU domain to bind LPL would be sufficient for LPL transport into capillaries. Also, it seemed possible that the ability of the AD to accelerate GPIHBP1-LPL binding kinetics would be inconsequential in vivo because of surplus capacity in the system. An approximately 75% decrease in GPIHBP1 expression in mice had no effect on plasma TG metabolism — even in mice fed a high-fat diet (22).

To test the in vivo relevance of GPIHBP1’s AD in plasma TG metabolism, we created a mutant-*Gpihbp1* mouse lacking the long stretch of acidic residues as well as the tyrosine that was sulfated in human GPIHBP1 (8). The mutant mice had moderate hypertriglyceridemia, but the mechanism was highly unexpected. With a combination of physiologic studies in mice, high-resolution immunofluorescence microscopy, and advanced biophysical analyses, we showed that the AD is essential for the transit of GPIHBP1-LPL complexes from the abluminal to the luminal surface of capillary ECs. In the absence of the AD, LPL binds to GPIHBP1 on the abluminal surface of ECs, but the GPIHBP1-LPL complexes remain trapped in that location by persistent electrostatic interactions between LPL’s basic patch and abluminal HSPGs. Trafficking of the mutant GPIHBP1 across ECs was normalized by disrupting electrostatic interactions with heparin or an AD peptide. Thus, a crucial function of the AD is to sheath LPL’s basic patch, abrogating abluminal LPL-HSPG electrostatic interactions and freeing GPIHBP1-LPL complexes to move across ECs to the capillary lumen.

## Results

### Mouse models to study the function of GPIHBP1’s AD.

To understand the in vivo functional relevance of GPIHBP1’s AD, we first created a mutant allele, *Gpihbp1*^D^, with a deletion of exon 2 sequences encoding the long stretch of acidic residues and the adjacent tyrosine (Supplemental Figure 1, A and B; supplemental material available online with this article; https://doi.org/10.1172/JCI157500DS1). The allele was verified by DNA sequencing, but Gpihbp1 transcripts in *Gpihbp1*^D/D^ mice were extremely low and there was skipping of exon 2 (Supplemental Figure 1, C–E). GPIHBP1 protein was undetectable (Supplemental Figure 1F), and the mice had chylomicronemia (Supplemental Figure 1G). We next created a mutant allele, *Gpihbp1*^A^, in which most of the acidic amino acids were replaced with alanine (Supplemental Figure 2, A and B). Again, *Gpihbp1* transcript levels were extremely low (Supplemental Figure 2C), GPIHBP1 protein was undetectable (Supplemental Figure 2D), and *Gpihbp1*^A/A^ mice had chylomicronemia. While these mouse models underscored the crucial role of GPIHBP1 in plasma TG metabolism, they were not helpful for elucidating the functional relevance of the AD.

**Figure 1:**
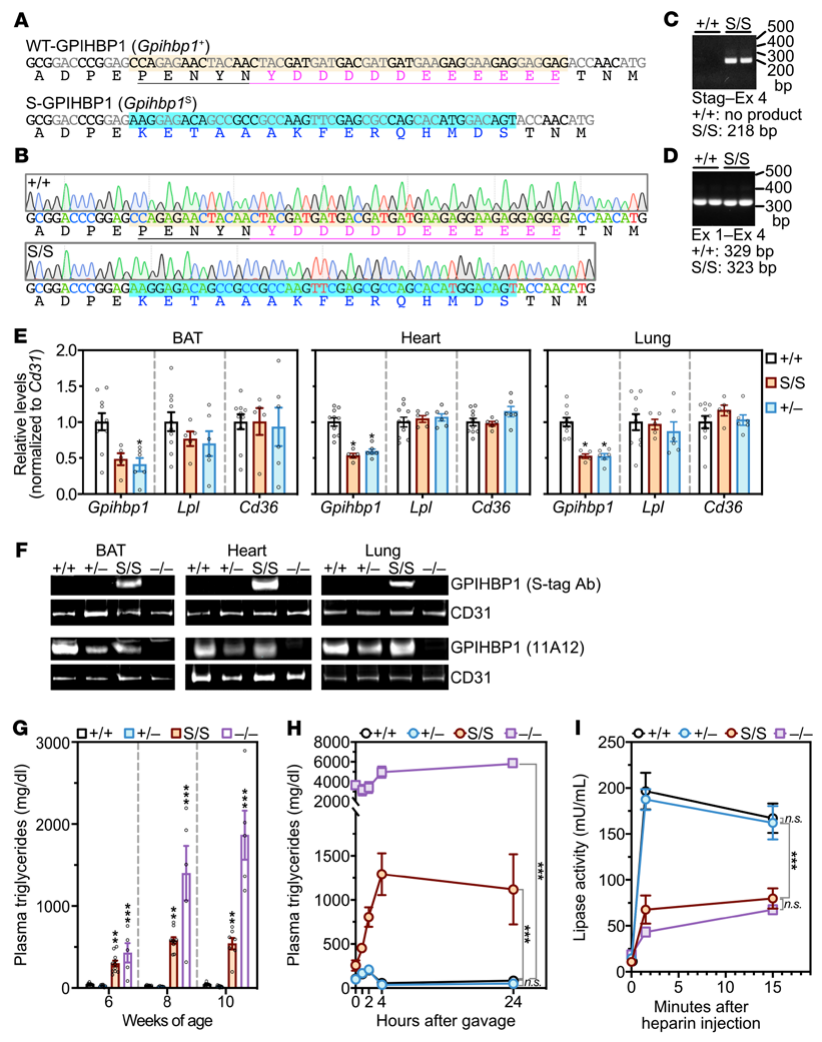
A mutant *Gpihbp1* allele in which the sulfated tyrosine and long stretch of acidic residues were replaced with an S-protein tag. (**A**) Nucleotide and amino acid sequences for the WT allele (*Gpihbp1*^+^) and an S-protein mutant allele (*Gpihbp1*^S^). In *Gpihbp1*^S^, 51 nucleotides (highlighted salmon in the WT allele) were replaced by 45 nucleotides encoding an S-protein tag (nucleotides and amino acids in the *Gpihbp1*^S^ allele are blue). In the *Gpihbp1*^+^ allele, the stretch of residues that were changed in the *Gpihbp1*^S^ allele are underlined; the long stretch of acidic residues (and the immediately adjacent sulfated tyrosine) are magenta. (**B**) DNA sequencing chromatograms from *Gpihbp1*^+/+^ and *Gpihbp1*^S/S^ mice. (**C**) PCR products from heart cDNA (with the forward primer in the S-protein sequences and the reverse primer in *Gpihbp1* exon 4). (**D**) PCR products from heart cDNA (with the forward primer in exon 1 and the reverse primer in exon 4). A single DNA product was amplified (no exon 2 skipping). (**E**) *Gpihbp1*, *Lpl*, and *Cd36* transcript levels (normalized to *Cd31* expression) in brown adipose tissue (BAT), heart, and lung, as judged by qRT-PCR. *n* = 10 *Gpihbp1*^+/+^, *n* = 5 *Gpihbp1*^S/S^, *n* = 6 *Gpihbp1*^+/–^ mice. *P < 0.05. (**F**) Western blots of BAT, heart, and lung extracts in *Gpihbp1*^+/+^, *Gpihbp1*^+/–^, *Gpihbp1*^S/S^, and *Gpihbp1*^–/–^ mice with a CD31-specific antibody and either an S-protein (S-tag) antibody (first row) or the GPIHBP1-specific antibody 11A12 (third row). Quantification of GPIHBP1 bands in 4 independent Western blots is shown in Figure 6D. (**G**) Plasma triglyceride levels in male *Gpihbp1*^+/+^, *Gpihbp1*^+/–^, and *Gpihbp1*^S/S^ mice at 6, 8, and 10 weeks of age (*n* = 5–11 mice/group and time point). ***P < 0.001 for *Gpihbp1*^+/+^ vs. *Gpihbp1*^–/–^ and *Gpihbp1*^+/–^ vs. *Gpihbp1*^–/–^; **P < 0.01 for *Gpihbp1*^+/+^ vs. *Gpihbp1*^S/S^ and *Gpihbp1*^+/–^ vs. *Gpihbp1*^S/S^. (**H**) Plasma triglyceride levels at baseline and 1, 2, 4, and 24 hours after an intragastric bolus of 100 μL of corn oil in 10-week-old male *Gpihbp1*^+/+^ (*n* = 10), *Gpihbp1*^S/S^ (*n* = 7), *Gpihbp1*^+/–^ (*n* = 6), and *Gpihbp1*^–/–^ (*n* = 3) mice. (**I**) Triglyceride hydrolase activity in the plasma of *Gpihbp1*^+/+^ (*n* = 6), *Gpihbp1*^+/–^ (*n* = 6), *Gpihbp1*^S/S^ (*n* = 6), and *Gpihbp1*^–/–^ (*n* = 3) mice before (time 0) and 1.5 and 15 minutes after an injection of heparin (15 U). NS, not significant. ***P < 0.001. Means were compared using a 2-way ANOVA test in panels **E** and **G**–**I**.

We next created a mutant allele, *Gpihbp1*^S^, in which the long stretch of acidic residues and the adjacent tyrosine were replaced with an S-protein tag (Figure 1, A–D). Transcript levels were approximately half that of WT, similar to those in heterozygous *Gpihbp1*-knockout mice (*Gpihbp1*^+/–^) (Figure 1E). Western blots of tissue extracts documented robust expression of the mutant GPIHBP1 (S-GPIHBP1) in *Gpihbp1*^S/S^ mice (more than in *Gpihbp1*^+/–^ mice; Figure 1F). Fasting TG levels in chow-fed *Gpihbp1*^S/S^ mice were elevated (~500 mg/dL), whereas they were very low in *Gpihbp1*^+/–^ and *Gpihbp1*^+/+^ mice (Figure 1G). Following an intragastric oil bolus, TG levels increased sharply in *Gpihbp1*^S/S^ mice (Figure 1H). LPL activity levels in postheparin plasma were lower in *Gpihbp1*^S/S^ mice than in *Gpihbp1*^+/–^ or *Gpihbp1*^+/+^ mice (Figure 1I).

### S-GPIHBP1 reaches the surface of ECs and binds LPL in a stable fashion.

We expected that the S-protein mutation would not affect attachment of S-GPIHBP1 to the cell surface because the AD is distant from the C-terminal sequences that trigger the addition of the GPI anchor. Indeed, when S-GPIHBP1 was expressed in CHO cells, it reached the cell surface and was releasable with phosphatidylinositol-specific phospholipase C (Supplemental Figure 3, A and B). We also expected that S-GPIHBP1 would reach the luminal surface of capillary ECs in vivo. Indeed, when hearts of *Gpihbp1*^S/+^ mice were perfused with an S-protein–specific antibody, the antibody bound to S-GPIHBP1 along the luminal surface of capillaries (Supplemental Figure 3C).

The AD in human GPIHBP1 contains a sulfated tyrosine adjacent to the long stretch of acidic residues (8). WT mouse GPIHBP1 contains 2 tyrosines in this region (Tyr^35^ and Tyr^37^). To determine whether those tyrosines are sulfated, we analyzed WT-GPIHBP1 (purified from *Drosophila* S2 cells) by mass spectrometry. One half of the WT-GPIHBP1 molecules contained 2 sulfated tyrosines, while the other half had only 1 sulfated tyrosine (Supplemental Figure 4). Neither tyrosine is present in S-GPIHBP1 (Supplemental Figure 4).

Because the LU domain in S-GPIHBP1 is intact, we suspected that it would retain the capacity to bind LPL. Indeed, LPL bound to S-GPIHBP1–expressing CHO cells, but there was no binding to cells that expressed a mutant GPIHBP1 (W108S-GPIHBP1) harboring a single amino acid substitution in the LU domain that disrupts the GPIHBP1-LPL binding interface (ref. 12 and Supplemental Figure 5). Stable binding of LPL to S-GPIHBP1 was also evident in SPR experiments. In those studies, we compared the abilities of WT-GPIHBP1 and S-GPIHBP1 to bind to LPL that was immobilized on the surface of sensor chips. At a physiologic NaCl concentration (150 mM), the on-rate for S-GPIHBP1 binding to LPL was significantly lower than for WT-GPIHBP1 (Supplemental Figure 6). Once bound, however, the binding of S-GPIHBP1 to LPL was stable (i.e., off-rates for S-GPIHBP1 and WT-GPIHBP1 were similar; Supplemental Figure 6). The differences in the on-rate for S-GPIHBP1 and WT-GPIHBP1 binding to LPL were markedly reduced at a higher NaCl concentration (750 mM; Supplemental Figure 6), indicating that the differences in binding kinetics at the physiologic NaCl concentration were driven by differences in electrostatic interactions.

### Low levels of LPL in capillaries of Gpihbp1^S/S^ mice.

**Figure 2:**
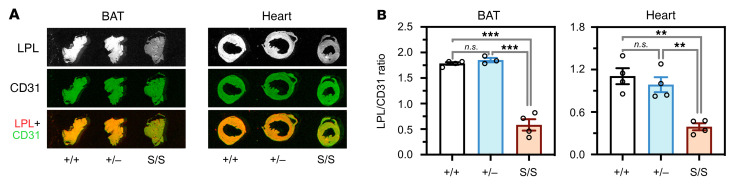
Reduced amounts of LPL on the luminal surface of blood vessels in *Gpihbp1*^S/S^ mice. IRDye 680–3174 IgG (antibody against mouse LPL) and IRDye 800–2H8 (antibody against CD31) were injected intravenously into *Gpihbp1*^+/+^, *Gpihbp1*^+/–^, and *Gpihbp1*^S/S^ mice. After 3 minutes, mice were perfused extensively with PBS and perfusion-fixed. (**A**) Representative images of the binding of antibodies 3174 and 2H8 to sections of brown adipose tissue (BAT) and heart. (**B**) The intensity of the IRDye 680 signal (reflecting LPL antibody binding) and the IRDye 800 signal (reflecting CD31 antibody binding) were quantified, and the LPL/CD31 ratios in BAT and heart sections were calculated. *n* = 4 *Gpihbp1*^+/+^, *n* = 3 *Gpihbp1*^+/–^, and *n* = 4 *Gpihbp1*^S/S^ mice for BAT. *n* = 4 mice/genotype for heart. Ten sections/mouse were analyzed. NS, not significant. **P < 0.01; ***P < 0.001. A 1-way ANOVA test was used to compare means in panel **B**.

The LPL released into plasma after an injection of heparin is generally assumed to originate from the luminal surface of capillaries. While we had observed low levels of postheparin LPL activity in *Gpihbp1*^S/S^mice (Figure 1I), we wanted to assess intravascular LPL stores more directly. We therefore injected an IRDye 680–labeled mouse LPL antibody (no. 3174) and an IRDye 800–labeled CD31 antibody (2H8) into the tail vein of *Gpihbp1*^+/+^, *Gpihbp1*^+/–^, and *Gpihbp1*^S/S^ mice. After 3 minutes, the mice were extensively perfused with PBS, perfusion-fixed, sections of brown adipose tissue (BAT) and heart were prepared, and the IRDye 800 and IRDye 680 signals in tissue sections were measured. These studies revealed that LPL stores within capillaries, relative to CD31, were lower in *Gpihbp1*^S/S^ mice than in *Gpihbp1*^+/+^ or *Gpihbp1*^+/–^ mice (Figure 2).

### Increased amounts of LPL on capillaries of Gpihbp1^S/S^ mice.

**Figure 3:**
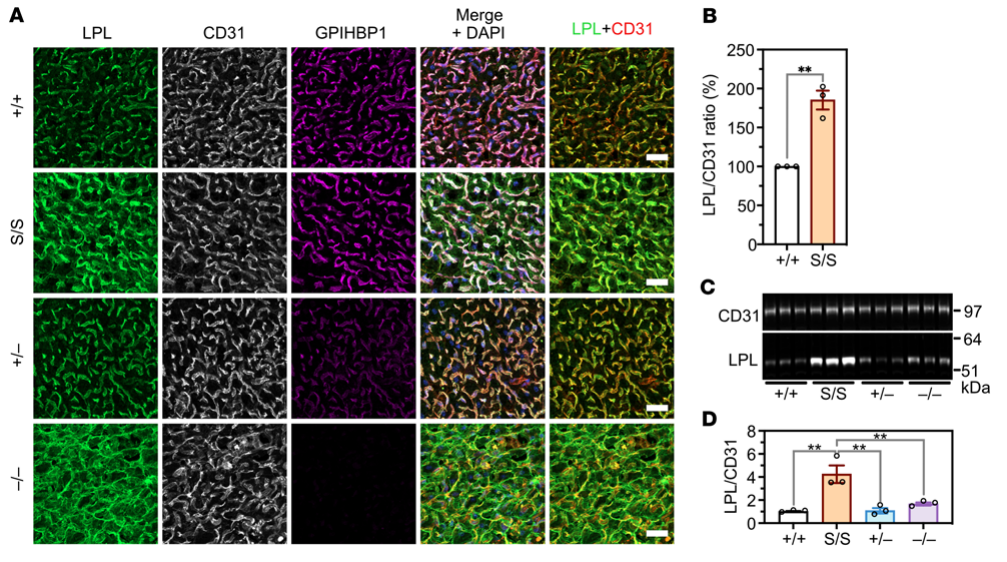
LPL, CD31, and GPIHBP1 expression in interscapular brown adipose tissue (BAT) of *Gpihbp1*^+/+^, *Gpihbp1*^S/S^*, Gpihbp1*^+/–^, and *Gpihbp1*^–/–^ mice. (**A**) Confocal immunofluorescence studies of LPL, CD31, and GPIHBP1 in BAT from *Gpihbp1*^+/+^, *Gpihbp1*^S/S^, *Gpihbp1*^+/–^, and *Gpihbp1*^–/–^ mice. Scale bars: 20 μm. (**B**) LPL/CD31 fluorescence intensity ratios in BAT capillaries of *Gpihbp1*^+/+^ and *Gpihbp1*^S/S^ mice. Fluorescence intensity ratios were quantified in 3 independent experiments (>100 capillaries/genotype). The ratio in *Gpihbp1*^S/S^ capillaries in each experiment was normalized to the ratio in *Gpihbp1*^+/+^ capillaries (set at 1.0). (**C**) Western blot studies of CD31 and LPL in BAT extracts (*n* = 3 mice/group). Each lane represents an individual mouse. (**D**) LPL/CD31 ratios in BAT extracts, as judged by quantification of the intensity of LPL and CD31 bands in panel **C**. The LPL/CD31 intensity ratio was higher in *Gpihbp1*^S/S^ mice than in the other groups of mice. **P < 0.01. Means were compared using a 2-tailed Student’s *t* test in panel **B**, and a 1-way ANOVA test in panel **D**.

To assess GPIHBP1-LPL interactions in vivo, we imaged LPL, CD31, and GPIHBP1 in BAT by immunofluorescence microscopy (Figure 3A). In *Gpihbp1*^+/+^ mice, LPL was located mainly on capillaries (colocalizing with GPIHBP1 and CD31), but small amounts were detected outside of capillaries (in parenchymal cells or the interstitial spaces). LPL was also located on capillaries in *Gpihbp1*^+/–^ mice (where GPIHBP1 staining was less intense). In *Gpihbp1*^–/–^ mice, LPL was distributed diffusely throughout the interstitial spaces, such that it was virtually impossible to discern LPL association with capillaries. In *Gpihbp1*^S/S^ mice, LPL was located mainly on capillary ECs, but some LPL was present outside of capillaries (Figure 3A). Of note, LPL staining of BAT capillaries was more intense in *Gpihbp1*^S/S^ mice than in *Gpihbp1*^+/+^ or *Gpihbp1*^+/–^ mice (Figure 3A). In 3 independent experiments, LPL/CD31 fluorescence intensity ratios on capillary ECs were 91.3%, 102.4%, and 61.9% higher in *Gpihbp1*^S/S^ mice than in *Gpihbp1*^+/+^ mice (Figure 3B). Consistent with that finding, amounts of LPL in BAT extracts were greater in *Gpihbp1*^S/S^ mice than in *Gpihbp1*^+/+^ mice (Figure 3, C and D). Similarly, LPL staining of heart capillaries was more intense in *Gpihbp1*^S/S^ mice than in *Gpihbp1*^+/+^ mice (Supplemental Figure 7, A and B), and amounts of LPL in heart extracts were greater in *Gpihbp1*^S/S^ mice than in *Gpihbp1*^+/+^ mice (Supplemental Figure 7, C and D).

### The LPL on capillaries of Gpihbp1^S/S^ mice is trapped on the abluminal surface of ECs.

**Figure 4:**
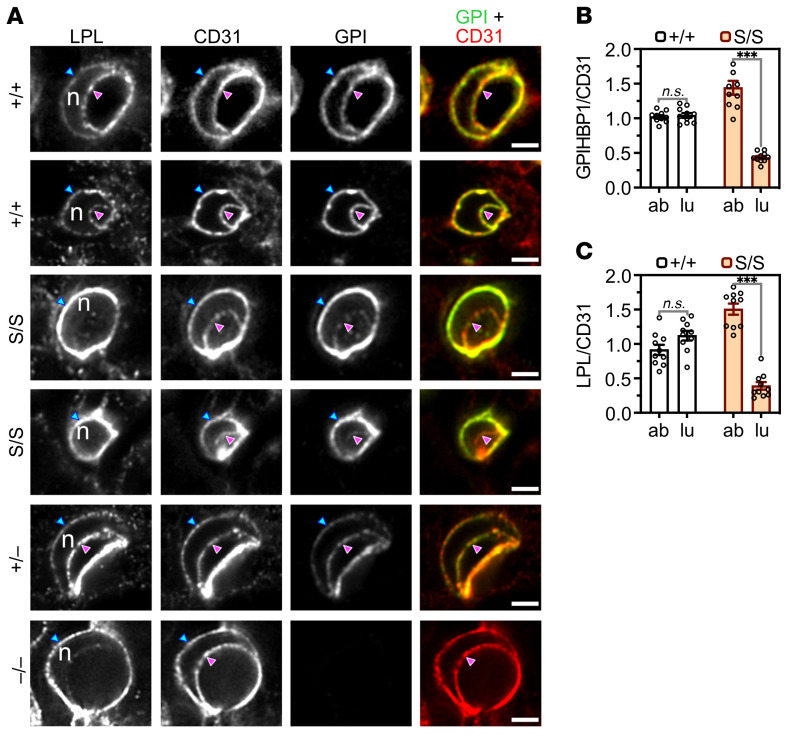
High-magnification confocal microscopy to assess distributions of S-GPIHBP1 and mouse LPL on the abluminal plasma membrane (APM) and luminal plasma membrane (LPM) of capillary endothelial cells in brown adipose tissue (BAT). (**A**) LPL, CD31, and GPIHBP1 expression in cross sections of BAT capillaries from *Gpihbp1*^+/+^, *Gpihbp1*^S/S^, *Gpihbp1*^+/–^, and *Gpihbp1*^–/–^ mice. The presence of an endothelial cell nucleus (n) separates the APM (blue arrowhead) from the LPM (magenta arrowhead). S-GPIHBP1 was distributed asymmetrically between the APM and LPM, with greater amounts on the APM. Amounts of LPL on the LPM were low in *Gpihbp1*^S/S^ mice. Scale bars: 2 μm. GPI, GPIHBP1. (**B** and **C**) GPIHBP1/CD31 (**B**) and LPL/CD31 (**C**) fluorescence intensity ratios in the APM and LPM of BAT capillary endothelial cells in *Gpihbp1*^+/+^ and *Gpihbp1*^S/S^ mice (*n* = 10 capillary cross sections/group). Ratios in the APM and LPM were normalized to the ratio in the “double plasma membrane” segment of the cross section (set at 1.0) where the APM and LPM are not separated by a cell nucleus. ab, APM; lu, LPM. NS, not significant. ***P < 0.001. Means were compared with a 2-tailed Student’s *t* test in panels **B** and **C**.

The microscopy observations posed a conundrum: Why were there greater amounts of LPL on capillary ECs in *Gpihbp1*^S/S^ mice but lower amounts within the capillary lumen? To address this issue, we imaged the distribution of LPL, GPIHBP1, and CD31 in BAT capillary cross sections containing an EC nucleus (Figure 4A). The presence of the nucleus made it possible to resolve the abluminal plasma membrane (APM) of ECs from the luminal plasma membrane (LPM) (2). In *Gpihbp1*^+/+^ and *Gpihbp1*^+/–^ mice, WT-GPIHBP1 was distributed evenly between the APM and the LPM (Figure 4A). In *Gpihbp1*^S/S^ mice, S-GPIHBP1 was located mainly on the APM, with only trace amounts on the LPM (Figure 4A). In *Gpihbp1*^+/+^ mice, the GPIHBP1/CD31 fluorescence intensity ratios on the APM and LPM of ECs were similar, consistent with an even distribution of WT-GPIHBP1 between the APM and LPM (Figure 4B). In *Gpihbp1*^S/S^ mice, the GPIHBP1/CD31 ratio was high (~1.5) on the APM but low (~0.5) on the LPM (Figure 4B), reflecting the fact that most of the S-GPIHBP1 was located on the APM. Similarly, the LPL/CD31 fluorescence intensity ratio was high on the APM of ECs of *Gpihbp1*^S/S^ mice (~1.5) but low (~0.4) on the LPM (Figure 4C).

**Figure 5:**
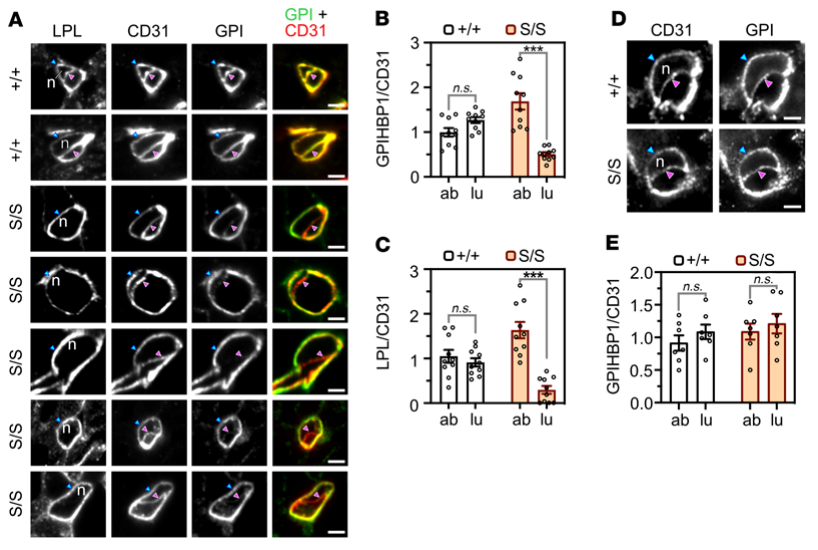
High-magnification confocal microscopy to assess distributions of GPIHBP1 and LPL on the abluminal plasma membrane (APM) and luminal plasma membrane (LPM) of heart and lung capillary endothelial cells. (**A**) LPL, CD31, and GPIHBP1 expression in cross sections of heart capillaries from *Gpihbp1*^+/+^ and *Gpihbp1*^S/S^ mice. The endothelial cell nucleus (n) separates the APM (blue arrowhead) from the LPM (magenta arrowhead). S-GPIHBP1 was distributed asymmetrically between the APM and LPM, with greater amounts on the APM. (**B** and **C**) GPIHBP1/CD31 (**B**) and LPL/CD31 (**C**) fluorescence intensity ratios in the APM and LPM of heart capillary endothelial cells (*n* = 10 capillary cross sections/group). Ratios in the APM and LPM were normalized to the ratio in the “double plasma membrane” segment (set at 1.0) where the APM and LPM are not separated by a nucleus. (**D**) Confocal micrographs of GPIHBP1 and CD31 expression in lung capillaries from *Gpihbp1*^+/+^ and *Gpihbp1*^S/S^ mice. Each cross section contains an endothelial cell nucleus (n), making it possible to resolve the APM (blue arrowhead) from the LPM (magenta arrowhead). (**E**) GPIHBP1/CD31 fluorescence intensity ratios in the APM and LPM of lung capillary endothelial cells (*n* = 7 capillary cross sections/group). Scale bars: 2 μm. GPI, GPIHBP1. ab, APM; lu, LPM. NS, not significant. ***P < 0.001. A 2-tailed Student’s *t* test was used to compare means in panels **B**, **C**, and **E**.

Consistent findings were observed in the heart. In *Gpihbp1*^+/+^ mice, GPIHBP1 was distributed evenly between the APM and the LPM of ECs, whereas in *Gpihbp1*^S/S^ mice there was an accumulation of S-GPIHBP1 on the APM (Figure 5A). In *Gpihbp1*^+/+^ heart ECs, the GPIHBP1/CD31 and LPL/CD31 fluorescence intensity ratios were similar on the APM and the LPM (Figure 5, B and C). In *Gpihbp1*^S/S^ heart ECs, the GPIHBP1/CD31 ratio was 3-fold higher on the APM than on the LPM (Figure 5B), and the LPL/CD31 ratio was 5-fold higher on the APM than on the LPM (Figure 5C).

We suspected that the accumulation of S-GPIHBP1 on the APM might be due to persistent electrostatic interactions between the abluminal S-GPIHBP1–bound LPL and cell-surface HSPGs. If that were the case, we reasoned that S-GPIHBP1 would be distributed evenly between the APM and LPM of ECs in the lung (where LPL expression is negligible) (Supplemental Figure 8). Indeed, S-GPIHBP1 was distributed evenly between the APM and the LPM in lung capillary ECs (Figure 5, D and E).

**Figure 6:**
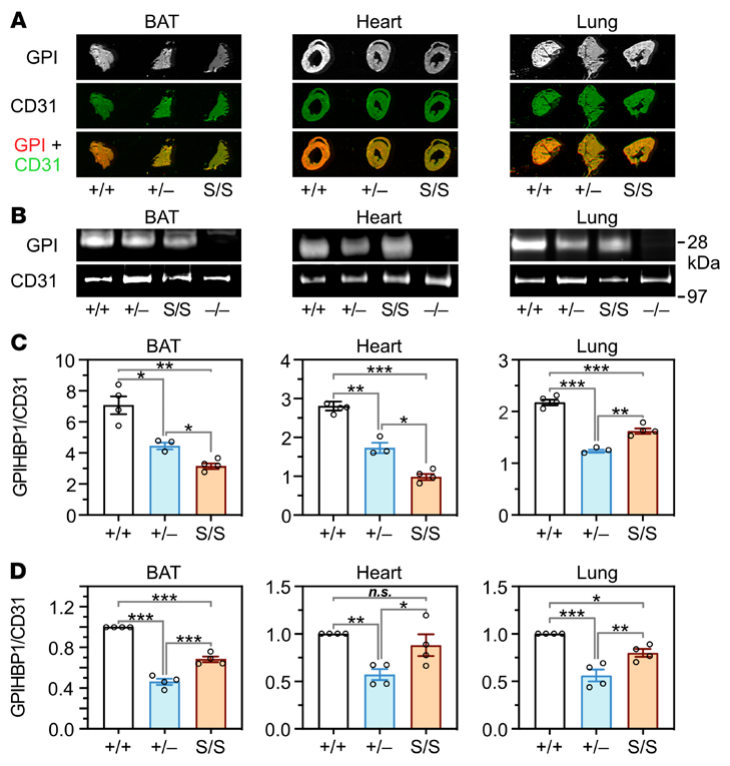
Assessing amounts of GPIHBP1, relative to CD31, along the capillary lumen in tissue sections as well as the amounts of GPIHBP1, relative to CD31, in whole tissue extracts. (**A**) Representative images of tissue sections that show the binding of an IRDye 800–labeled GPIHBP1 antibody (11A12; red) and an IRDye 680–labeled CD31 antibody (2H8; green) to the luminal surface of blood vessels in brown adipose tissue (BAT), heart, and lung of *Gpihbp1*^+/+^, *Gpihbp1*^+/–^, and *Gpihbp1*^S/S^ mice. GPI, GPIHBP1. (**B**) Representative Western blots (with antibodies 11A12 and 2H8) of BAT, heart, and lung extracts from *Gpihbp1*^+/+^, *Gpihbp1*^+/–^, *Gpihbp1*^S/S^, and *Gpihbp1*^–/–^ mice. (**C**) GPIHBP1/CD31 ratios (calculated from IRDye 800 and IRDye 680 signals) in sections of BAT, heart, and lung from *Gpihbp1*^+/+^ (*n* = 4), *Gpihbp1*^+/–^ (*n* = 3), and *Gpihbp1*^S/S^ (*n* = 4) mice. Ten sections were analyzed in each tissue of each mouse. (**D**) Relative amounts of GPIHBP1 and CD31 in BAT, heart, and lung extracts from *Gpihbp1*^+/+^, *Gpihbp1*^+/–^, and *Gpihbp1*^S/S^ mice, as judged by quantification of Western blot bands (*n* = 4 independent experiments). NS, not significant; *P < 0.05; **P < 0.01; ***P < 0.001. Means were compared with 1-way ANOVA tests in panels **C** and **D**.

The microscopy studies revealed that S-GPIHBP1 was located mainly on the APM of ECs in BAT and heart (where LPL expression is high) but was distributed evenly between the APM and LPM of ECs in the lung (where LPL expression is negligible). To test these observations with an independent experimental system, we injected an IRDye 800–labeled GPIHBP1 antibody (11A12) and the IRDye 680–labeled CD31 antibody 2H8 into the tail vein of *Gpihbp1*^+/+^, *Gpihbp1*^+/–^, and *Gpihbp1*^S/S^ mice. After 3 minutes, the mice were perfused extensively, sections of BAT, heart, and lung were prepared, and the IRDye 800 and IRDye 680 signals in tissue sections were quantified. In BAT and heart, the IRDye 800/IRDye 680 ratio (reflecting relative amounts of GPIHBP1 and CD31 on the luminal surface of blood vessels) was lower in *Gpihbp1*^S/S^ mice than in *Gpihbp1*^+/–^ mice (Figure 6, A and C). However, when we assessed total amounts of GPIHBP1 and CD31 in BAT and heart extracts by Western blotting, the GPIHBP1/CD31 ratio was higher in *Gpihbp1*^S/S^ mice than in *Gpihbp1*^+/–^ mice (Figure 6, B and D). These findings are quite consistent with the microscopy studies, which had revealed low levels of S-GPIHBP1 on the luminal surface of capillaries (Figure 4, A and B, and Figure 5, A and B).

Both the IRDye 800/IRDye 680 ratio in lung sections (Figure 6, A and C) and the GPIHBP1/CD31 ratio in lung extracts (Figure 6, B and D) were higher in *Gpihbp1*^S/S^ mice than in *Gpihbp1*^+/–^ mice. Thus, unlike the situation in heart and BAT, intravascular levels of S-GPIHBP1 in the lung (depicted in Figure 6, A and C) were proportionate to the total amounts of S-GPIHBP1 in the lung (depicted in Figure 6, B and D). These findings, which reflect unimpeded transport of S-GPIHBP1 across lung ECs, are consistent with microscopy studies showing that S-GPIHBP1 is distributed evenly between the APM and LPL of lung capillary ECs (Figure 5, D and E).

### S-GPIHBP1 cannot release LPL from heparin-binding sites in vitro, explaining why S-GPIHBP1 is trapped on the abluminal surface of ECs in vivo.

**Figure 7:**
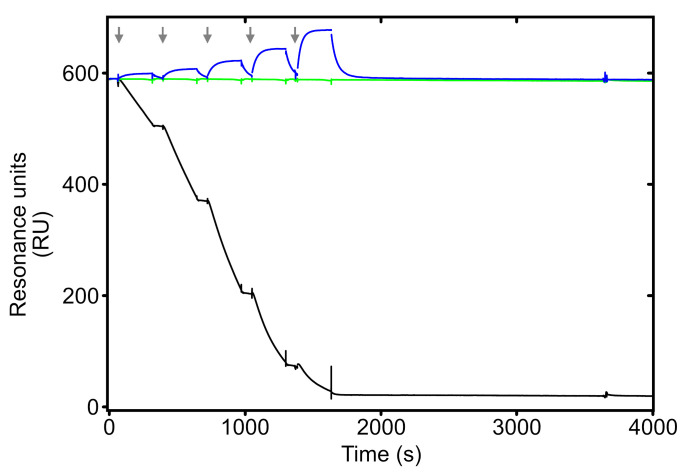
Assessing the ability of WT-GPIHBP1 and S-GPIHBP1 to detach LPL from heparin sulfate DP4 on Biacore sensor chips. Purified mouse LPL was adsorbed onto a Biacore CM5 sensor chip that had been coated with a high density of heparin sulfate DP4. LPL was stably attached, by electrostatic interactions, to the heparin sulfate DP4, evident by the nondecaying baseline upon the injection of buffer alone (green line). Injection of purified WT-GPIHBP1 resulted in a progressive loss of LPL (black line) into the buffer flow. Injections of mouse S-GPIHBP1 resulted in binding of S-GPIHBP1 to the HSPG-bound LPL, but there was no release of LPL into the buffer flow (blue line). Five consecutive injections of WT-GPIHBP1 or S-GPIHBP1 (serial dilutions from 12.5 nM to 200 nM) or buffer alone are indicated by the gray arrows.

As noted earlier, we suspected that the accumulation of S-GPIHBP1 on the APM of BAT and heart ECs resulted from electrostatic tethering of S-GPIHBP1–bound LPL to cell-surface HSPGs. We further suspected that a crucial physiologic function of the AD in WT mice is to abrogate those electrostatic interactions. To explore that concept, we used SPR to examine interactions of WT-GPIHBP1 and S-GPIHBP1 with LPL that had been immobilized (by electrostatic interactions) on sensor chips coated with high levels of a heparin fragment. When WT-GPIHBP1 was flowed over the sensor chip, it detached LPL from the heparin fragment (Figure 7). In contrast, when S-GPIHBP1 was flowed over the sensor chip, it simply bound to the LPL and failed to detach it (Figure 7).

### S-GPIHBP1 does not move to the LPM in living mice.

**Figure 8:**
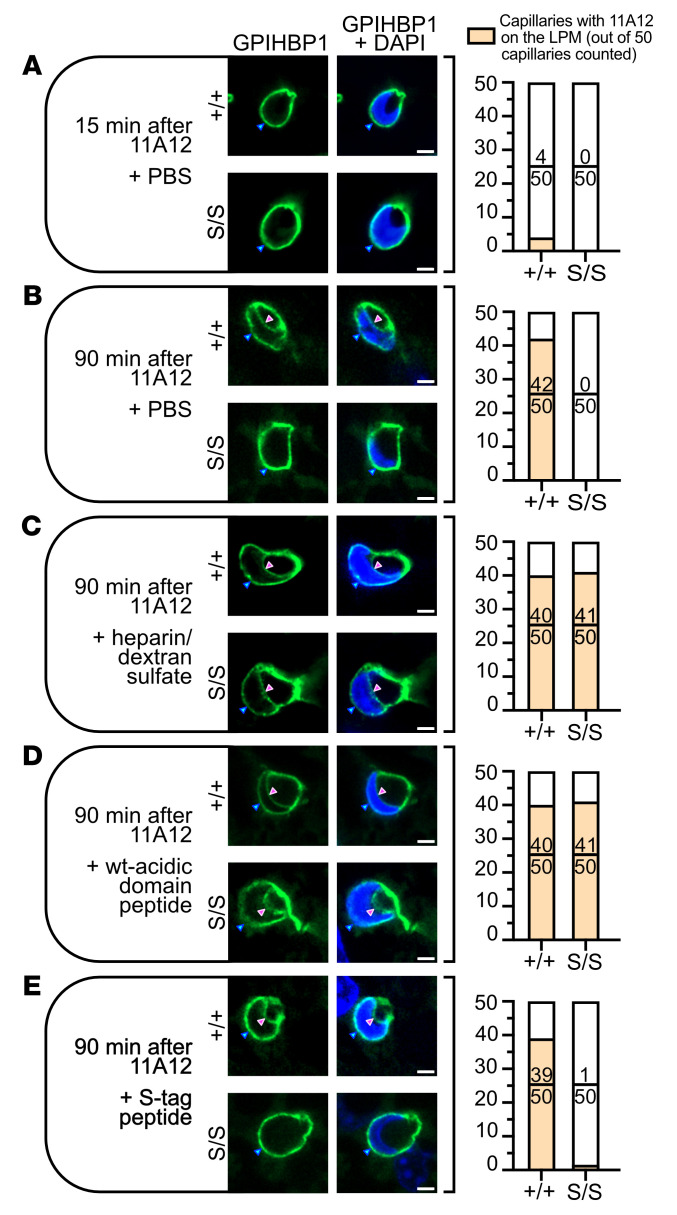
Assessing movement of the GPIHBP1-specific antibody 11A12 from the abluminal plasma membrane (APM) to the luminal plasma membrane (LPM) in brown adipose tissue (BAT) capillary endothelial cells of living mice. Alexa Fluor 488–11A12 (green) was injected into the interscapular BAT of *Gpihbp1*^+/+^ and *Gpihbp1*^S/S^ mice. After 15 or 90 minutes, images of capillary cross sections containing an endothelial cell nucleus (blue) were recorded by fluorescence microscopy. The presence of the cell nucleus made it possible to visualize 11A12 on the APM (blue arrowhead) and the LPM (magenta arrowhead). Shown here is a representative capillary cross section for each experimental condition. On the right, we show the number of capillary cross sections (from a total of 50 counted) in which Alexa Fluor 488–11A12 was detectable at the capillary lumen. Three additional cross sections per experimental condition are shown in Supplemental Figure 9. (**A** and **B**) Capillary cross sections in BAT from *Gpihbp1*^+/+^ and *Gpihbp1*^S/S^ mice 15 minutes (**A**) or 90 minutes (**B**) after the injection of Alexa Fluor 488–11A12. (**C**) Capillary cross sections in BAT 90 minutes after an injection of Alexa Fluor 488–11A12, 0.75 U heparin, and 15 μg dextran sulfate. (**D** and **E**) Capillary cross sections in BAT 90 minutes after an injection of Alexa Fluor 488–11A12 and 34 μmol of a synthetic peptide corresponding to the WT GPIHBP1 AD (EDGDADPEPENYNYDDDDDEEEEEE) (**D**) or the S-protein tag (KETAAAKFERQHMDS) (**E**). Scale bars: 2 μm.

The SPR data strongly supported the idea that S-GPIHBP1–bound LPL is trapped on the APM of ECs by electrostatic interactions with HSPGs. To investigate this concept, we examined GPIHBP1 trafficking from the APM to the LPM of BAT ECs in living mice. It was shown previously that when the GPIHBP1-specific antibody 11A12 is injected into the interscapular BAT pad of *Gpihbp1*^+/+^ mice, it binds to GPIHBP1 on the APM and within 120 minutes is transported by GPIHBP1 to the LPM (2). In the current study, we injected Alexa Fluor 488–11A12 into the BAT of *Gpihbp1*^+/+^ and *Gpihbp1*^S/S^ mice and monitored its appearance on the LPM of ECs 15 or 90 minutes later. In *Gpihbp1*^+/+^ mice, 11A12 was detected on the LPM in only 4 of 50 capillaries at the 15-minute time point (Figure 8A and Supplemental Figure 9A) but was detected in 42 of 50 capillaries at the 90-minute time point (Figure 8B and Supplemental Figure 9B). In *Gpihbp1*^S/S^ mice, 11A12 was visualized on the APM at the 15-minute and 90-minute time points, but none reached the LPM at either time point (Figure 8, A and B, and Supplemental Figure 9, A and B).

### Disrupting LPL-HSPG electrostatic interactions normalizes S-GPIHBP1 movement to the LPM of ECs in living Gpihbp1^S/S^ mice.

We speculated that the electrostatic interactions between LPL and abluminal HSPGs prevented movement of antibody 11A12 to the capillary lumen in *Gpihbp1*^S/S^ mice. We further suspected that 11A12 trafficking would be restored to normal by disrupting those electrostatic interactions. To explore this idea, dextran sulfate and heparin were coinjected with the Alexa Fluor 488–11A12 into the BAT pads of *Gpihbp1*^+/+^ and *Gpihbp1*^S/S^ mice. Dextran sulfate/heparin normalized 11A12 trafficking across ECs in *Gpihbp1*^S/S^ mice (Figure 8C and Supplemental Figure 9C). An AD peptide also normalized 11A12 transport across ECs in *Gpihbp1*^S/S^ mice (Figure 8D and Supplemental Figure 9D), whereas an S-protein peptide did not (Figure 8E and Supplemental Figure 9E).

### Reduced capacity of purified S-GPIHBP1 to preserve LPL structure and activity.

The trapping of S-GPIHBP1–LPL complexes on the APM of ECs in *Gpihbp1*^S/S^ mice helps to explain their low intravascular levels of LPL and their high plasma TG levels. However, we hypothesized that reduced LPL stability in *Gpihbp1*^S/S^ mice might also contribute to the high plasma TG levels. Biophysical studies with purified proteins supported this idea. Purified WT-GPIHBP1 markedly increased the thermal stability of mouse LPL, increasing the melting temperature (*T_m_*) from 34.5°C ± 0.5°C to 52.5°C ± 0.2°C (Supplemental Figure 10). In contrast, S-GPIHBP1 had only modest effects (increasing the *T_m_* to only 39.3°C ± 0.8°C) (Supplemental Figure 10). Also, WT-GPIHBP1 was quite effective in preserving the TG hydrolase activity of mouse LPL at room temperature, while the effects of S-GPIHBP1 on LPL activity were modest (Supplemental Figure 11).

### Reduced specific activity of LPL in the postheparin plasma of Gpihbp1^S/S^ mice.

The thermal stability and LPL catalytic activity studies with purified proteins supported the notion that the AD preserves LPL structure and activity, but the key issue was whether LPL specific activity was lower in *Gpihbp1*^S/S^ mice. To explore this issue, we obtained postheparin plasma of *Gpihbp1*^+/+^ and *Gpihbp1*^S/S^ mice, measured LPL mass and activity, and then calculated the specific activity of LPL in the postheparin plasma. The specific activity of heparin-releasable LPL was approximately 49% lower in *Gpihbp1*^S/S^ mice than *Gpihbp1*^+/+^ mice (*P* < 0.001; Supplemental Figure 12), consistent with biochemical studies (Supplemental Figures 10 and 11) showing that S-GPIHBP1 is less effective in preserving LPL structure and activity.

## Discussion

GPIHBP1’s main function is to capture the LPL secreted by parenchymal cells and shuttle it across ECs to the capillary lumen (2). GPIHBP1’s LU domain is crucial for this process, evident by an absence of intracapillary LPL in mice with a single amino acid substitution in the LU domain (17), but for years the biological relevance of GPIHBP1’s AD has remained uncertain. In the current study, we created mutant mice in which GPIHBP1’s AD was replaced with an S-protein tag (*Gpihbp1*^S/S^). We found, quite unexpectedly, that GPIHBP1’s AD is required for the trafficking of GPIHBP1-LPL complexes across ECs. In the BAT and heart of *Gpihbp1*^S/S^ mice, only small amounts of S-GPIHBP1 moves across ECs to the LPM. Instead, S-GPIHBP1 and its LPL cargo accumulate on the APM, trapped there by persistent electrostatic interactions between the S-GPIHBP1–bound LPL and cell-surface HSPGs. Trafficking of the GPIHBP1-specific antibody (11A12) from the APM to the LPM was robust in *Gpihbp1*^+/+^ mice but virtually undetectable in *Gpihbp1*^S/S^ mice. Of note, however, 11A12 trafficking across ECs in *Gpihbp1*^S/S^ mice was normalized when the electrostatic interactions between LPL and adjacent HSPGs were disrupted with heparin/dextran sulfate or an AD peptide. Thus, a key physiologic function of the AD is to abrogate the electrostatic interactions between GPIHBP1-bound LPL and abluminal HSPGs, freeing GPIHBP1-LPL complexes to move across ECs to the LPM. This concept was strongly supported by SPR studies. WT-GPIHBP1 readily detached LPL from heparin-binding sites on sensor chips, whereas S-GPIHBP1 was simply captured by the LPL and failed to detach it. Thus, WT-GPIHBP1, but not S-GPIHBP1, interferes with the electrostatic interactions between LPL and HSPGs.

Images of capillary cross sections from *Gpihbp1*^S/S^ mice revealed intense staining of LPL on the APM but low levels of LPL on the LPM. The low levels of LPL in the capillary lumen were confirmed by studies in which LPL-specific antibodies were injected intravenously into mice. In those studies, the binding of the LPL-specific antibodies to the luminal surface of blood vessels was approximately 70% lower in *Gpihbp1*^S/S^ mice than in *Gpihbp1*^+/+^ mice. Levels of LPL in the postheparin plasma were also reduced by approximately 70%. The entry of LPL into capillaries of *Gpihbp1*^S/S^ mice was markedly reduced but not completely absent, explaining why the plasma TG levels were lower in *Gpihbp1*^S/S^ mice than in *Gpihbp1*^–/–^ mice.

A role for GPIHBP1’s AD in preventing electrostatic interactions between LPL and abluminal HSPGs is consistent with the crystal structure of the GPIHBP1-LPL complex (5). An electrostatic surface potential map based on the crystal structure revealed a large basic patch on the surface of LPL, formed by the confluence of several arginine- and lysine-rich heparin-binding motifs (5). LPL’s basic patch is distant from both its catalytic pocket and the hydrophobic surface that interfaces with GPIHBP1’s LU domain. GPIHBP1’s AD was not visualized in the crystal structure but was positioned to project over and interact, by electrostatic forces, with LPL’s basic patch. The sheathing of the basic patch by GPIHBP1’s AD would be expected to prevent interactions between LPL and cell-surface HSPGs. In contrast, when S-GPIHBP1 binds to LPL, LPL’s basic patch is left exposed and is free to bind to adjacent HSPGs.

In *Gpihbp1^–/–^* mice, where there is little or no LPL transport into capillaries, LPL is trapped on the surface of parenchymal cells and ECs, a result of electrostatic interactions between LPL’s basic patch and negatively charged HSPGs. In WT mice, GPIHBP1’s AD helps to ensnare LPL. By projecting more than 60 Å from the cell surface, the AD functions to “lasso” HSPG-bound LPL and bring it into stable interactions with the LU domain (8, 23). Once bound, the dissociation of LPL from GPIHBP1 is minimally affected by the presence of the AD. Aside from ensnaring interstitial LPL, the current studies show that the AD serves as a sheath for LPL’s basic patch, abrogating LPL interactions with cell-surface HSPGs and freeing GPIHBP1-LPL complexes to move to the LPM of ECs.

LPL was identified in the 1950s as an intravascular, heparin-releasable TG hydrolase (24, 25). By the early 1980s, the binding of LPL to the surface of cells was shown to be mediated by HSPGs (26, 27). For the next 3 decades, diagrams of plasma TG metabolism invariably depicted the binding of LPL to HSPGs on the luminal surface of blood vessels. After the discovery of GPIHBP1’s role in TG metabolism (1, 2), the focus shifted to GPIHBP1-LPL interactions and HSPGs were often omitted or downplayed in diagrams of TG metabolism (28–30). Now the tide has turned again, with a renewed appreciation of HSPGs in LPL/GPIHBP1 physiology. For example, Sundberg and coworkers discovered that LPL binding to an HSPG (syndecan-1) in exocytic vesicles is important for LPL secretion from cells (31, 32). Also, in the absence of GPIHBP1, HSPGs prevent the escape of LPL into the bloodstream (2). In the current study, we show that the prevention of LPL-HSPG interactions by GPIHBP1’s AD is required for trafficking of GPIHBP1-LPL complexes across capillary ECs. The ability of HSPGs to influence receptor-ligand interactions has been recognized for years (33–35), but the role of HSPGs in intravascular lipolysis stands as one of the best understood, in part because GPIHBP1-LPL interactions are accessible to both biophysical analyses (20) and experimental physiology studies in mouse models (1, 2, 4, 17).

The trapping of S-GPIHBP1–LPL complexes on the APM of ECs contributes to elevated plasma TG levels in *Gpihbp1*^S/S^ mice, but a second factor is reduced specific activity of LPL. HDX-MS studies revealed that LPL is susceptible to spontaneous loss of catalytic activity, a result of unfolding of LPL’s amino-terminal hydrolase domain (19, 36). The unfolding of LPL’s hydrolase domain can be inhibited by both full-length human GPIHBP1 and a human AD peptide but not by GPIHBP1’s LU domain alone (19, 36). In contrast, inhibition of ANGPTL4-catalyzed LPL unfolding requires full-length GPIHBP1 (36). In the current studies, we demonstrated that purified WT mouse GPIHBP1 preserves the structural integrity of purified mouse LPL, as judged by thermal stability studies. In contrast, the binding of S-GPIHBP1 had minimal effects on LPL thermal stability. Consistent with these findings, WT-GPIHBP1 was effective in preserving the TG hydrolase activity of mouse LPL, whereas the impact of S-GPIHBP1 was modest. The biochemical studies with purified proteins implied that GPIHBP1’s AD preserves LPL structure and activity, but the crucial issue was whether the AD is relevant to LPL catalytic activity in living mice. Our studies provided the answer, in that the specific activity of heparin-releasable LPL was approximately 49% lower in *Gpihbp1*^S/S^ mice than in *Gpihbp1*^+/+^ mice. It is possible that the reduced specific activity of the LPL in *Gpihbp1*^S/S^ mice reflects a reduced capacity of S-GPIHBP1 to protect LPL from ANGPTL4-catalyzed unfolding.

In conclusion, we show that GPIHBP1’s AD prevents electrostatic interactions between LPL and abluminal HSPGs, thereby allowing GPIHBP1-LPL complexes to move to the capillary lumen. GPIHBP1’s AD also plays a role in preserving LPL catalytic activity in vivo, evident by the reduced specific activity of LPL in *Gpihbp1*^S/S^ mice. The lower specific activity of LPL in *Gpihbp1*^S/S^ mice underscores the physiologic relevance of biophysical experiments with purified proteins, which had revealed a role for the AD in preserving the structural integrity of LPL (Supplemental Figure 10 and refs. 8, 19).

## Methods

See Supplemental Table 1 for details on antibodies, reagents, assays, and software.

### Genetically modified mice.

Mice (*Mus* *musculus*) with a deletion of the entire *Gpihbp1* gene (*Gpihbp1^–/–^*) were described previously (1); these mice had been backcrossed to C57BL/6 more than 6 times. L0-MCK mice (*Lpl^–/–^* mice with a human LPL transgene driven by the promoter of the muscle creatine kinase gene) were described previously (37). Mice were fed a chow diet and were housed in a barrier facility with a 12-hour light/12-hour dark cycle.

In association with the CRISPR/Cas9 Mouse Targeting Core at the University of Pennsylvania, we created a mutant *Gpihbp1* allele (*Gpihbp1*^S^) in which the sulfated tyrosine and long stretch of acidic residues (DDDDDEEEEEE) was replaced with an S-protein tag (KETAAAKFERQHMDS) (Figure 1). C57BL/6 zygotes were injected with 10 ng Cas9 transcript, a guide RNA (GAAATTAATACGACTCACTATAGGGAGATCATCGTAGTTGTAGTTCTCGTTTTAGAGCT), and a 171-bp double-stranded repair template (ACTCATGTCCCTGTGACACCAGGGAGTGGCTGGGCACAAGAAGATGGTGATGCGGACCCGGAGaaggagacagccgccgccaagttcgagcgccagcacatggacagtACCAACATGATCCCTGGAAGCAGGGACAGAGGTACCCCAGCTGAGGGCCCAGCTTCCTGCTCT; mutant sequence in lowercase). The repair fragment was designed to replace 51 bp in the WT *Gpihbp1* allele (CCAGAGAACTACAACTACGATGATGACGATGATGAAGAGGAAGAGGAGGAG) with 45 bp (AAGGAGACAGCCGCCGCCAAGTTCGAGCGCCAGCACATGGACAGT) encoding the S-protein tag. Multiple founders (C57BL/6 strain) were obtained, and the fidelity of the modification was established by DNA sequencing. The mutant allele was identified by PCR with a mutant allele–specific forward primer 5′-GGGACAAGGAATAGACCTGAG-3′ and reverse primer 5′-GTGCTGGCGCTCGAACTT-3′ (yielding a 308-bp product); the WT allele was identified with forward primer 5′-GGGGAATTCTGTCTCCTTCC-3′ and reverse primer 5′-TCTTCATCATCGTCATCATCG-3′ (yielding a 216-bp product).

We also created a deletion allele, *Gpihbp1*^D^, designed to remove part of GPIHBP1’s AD (Supplemental Figure 1). We used guide RNA GAAATTAATACGACTCACTATAGGGAGATAGTTGTAGTTCTCTGGCTCGTTTTAGAGCT and a 128-bp repair template (TAAGTGGCCAAAGCTTACTCATGTCCCTGTGACACCAGGGAGTGGCTGGGCACAAGAAGATGGTACCAACATGATCCCTGGAAGCAGGGACAGAGGTACCCCAGCTGAGGGCCCAGCTTCCTGCTCTG). The repair template was designed to delete 66 bp from exon 2, thereby removing amino acids 27 to 48 (including the sulfated tyrosine and the long stretch of acidic residues).

We also created (in association with the Transgenic Gene Targeting Core at the Gladstone Institutes) an alanine-substituted allele (*Gpihbp1*^A^) designed to replace the majority of the acidic residues in the AD with Ala or Ser (Supplemental Figure 2). The guide RNA was TCATCGTAGTTGTAGTTCTCTGG; the double-stranded break in exon 2 was repaired with a 200-bp repair template (GTCCTCTGCATCTAAGTGGCCAAAGCTTACTCATGTCCCTGTGACACCAGggagtggctgggcacaagcagctggtgcagcagccccggcaccagctaactacaactacgctgctgctgcagctacagcagctgctgctgcaaccaacatgatccctggaagcagggaCAGAGGTACCCCAGCTGAGGGCCCAGCTTCCT; mutant sequence in lowercase). We obtained a single founder animal in which exon 2 nucleotides GAAGATGGTGATGCGGACCCGGAGCCAGAGAACTACAACTACGATGATGACGATGATGAAGAGGAAGAGGAGGAG had been replaced with GCAGCTGGTGATGCGGACCCGGAGCCAGCTAACTACAACTACGCTGCTGCTGCAGCTACAGCAGCTGCTGCTGCA, thereby modifying GPIHBP1 amino acids 24 to 48 and replacing most of the acidic residues in the AD.

### Antibodies.

We used a rat monoclonal antibody (11A12) against mouse GPIHBP1 (11) and a goat polyclonal antibody against mouse LPL (38). Rabbit polyclonal antibodies against mouse LPL (antibodies 3174 and 3175) were produced by immunizing rabbits with recombinant mouse LPL; IgG fractions were generated with a protein G–Sepharose column. A rat monoclonal antibody against mouse LPL (27A) was created by Immuno-Biological Laboratories in Gunma, Japan, after immunizing rats with mouse LPL. A hamster monoclonal antibody, 2H8 against CD31, was obtained from the Developmental Studies Hybridoma Bank at the University of Iowa. A goat polyclonal antibody against the S-protein tag and a rabbit polyclonal antibody against β-actin were purchased from Abcam and Novus Biologicals, respectively; a goat polyclonal antibody against CD31 was purchased from R&D Systems; a monoclonal antibody against the V5 epitope tag was purchased from Thermo Fisher Scientific. IRDye 680 and IRDye 800 secondary antibodies were purchased from LI-COR. Alexa Fluor–conjugated secondary antibodies were purchased from Thermo Fisher Scientific and Jackson ImmunoResearch.

### Recombinant mouse LPL and mouse GPIHBP1 proteins.

Mouse LPL was expressed in Drosophila S2 cells and purified to homogeneity by heparin–Sepharose chromatography (39). We also produced soluble versions of WT mouse GPIHBP1 (residues 23–198) and S-GPIHBP1 in *Drosophila* S2 cells. The recombinant GPIHBP1 proteins were secreted because they lacked the carboxyl-terminal signal peptide that triggers the addition of a GPI anchor. WT-GPIHBP1 and S-GPIHBP1 were purified by immunoaffinity chromatography with monoclonal antibody 11A12 (11). Low levels of GPIHBP1 oligomers were removed by size-exclusion chromatography with a Sepharose G75 column and a running buffer of 10 mM Na_2_HPO_4_, 136 mM NaCl (pH 7.4). This procedure yielded homogeneous and monodisperse GPIHBP1 preparations. The molecular masses of WT-GPIHBP1 and S-GPIHBP1 were analyzed on an electrospray ionization (ESI) Tri-Wave Ion Mobility mass spectrometer (Synapt G2) connected with a Waters HDX-Manager. Samples were desalted for 2 minutes in 500 μL/min 0.23% aqueous formic acid solution (solvent A) with an Agilent 1260 Infinity quaternary pump (Agilent). Proteins were eluted from a 1.0 mm × 5 mm MassPREP Micro Desalting Column by a gradient flow provided by a nanoAcquity ultra-performance liquid chromatography Binary Solvent Manager (5%–50% gradient of 0.23% formic acid in pure acetonitrile; solvent B) over 3 minutes and 50%–90% solvent B over 1 minute at 50 μL/min flow rate. Data were analyzed with MassLynx software (Waters) using the Maximum Entropy deconvolution algorithm MaxEnt1, with an output mass range of 20,000–25,000 Da, a resolution of 1 Da/channel, and a uniform Gaussian model with peak width at half height at 0.8 Da.

### qRT-PCR.

RNA was extracted from tissues with TRI reagent (Molecular Research); cDNA was generated with random primers, oligo(dT), and SuperScript III (Invitrogen). qRT-PCR was performed in triplicate on a QuantStudio 5 Real-Time PCR System from Applied Biosystems with the SYBR Green PCR Master Mix (Bioland). Gene expression was calculated with the comparative C_T_ method (40). Primer details are provided in Supplemental Table 2.

### Western blots.

Plasma proteins and tissue extracts were size fractioned in 12% Bis-Tris SDS-polyacrylamide gels and transferred to a nitrocellulose membrane. Western blots were performed with antibody 11A12 (4 μg/mL), the S-protein tag antibody (2.5 μg/mL), the antibody against β-actin (5 μg/mL), or the polyclonal antibody against CD31 (0.5 μg/mL), followed by IRDye 680– or IRDye 800–labeled secondary antibodies (1:2,000). Antibody binding was quantified with an Odyssey infrared scanner (LI-COR). See the complete unedited blots in the supplemental material.

### Measurements of TGs and LPL mass and activity.

TG levels in mouse plasma samples were measured with the Serum Triglyceride Determination Kit (MilliporeSigma). For TG hydrolase activity measurements, we obtained baseline plasma samples (preheparin) as well as plasma samples 2 and 15 minutes after an intravenous injection of 15 U of heparin (postheparin). Blood samples were collected from the retroorbital sinus. TG hydrolase activity in fresh plasma samples was measured with a [^3^H]triolein substrate, and rat serum was used as a source of APOC2 (1, 41). To avoid aggregation of lipases, the plasma samples were initially adjusted to 1.2 M NaCl and 50 U/mL heparin. TG hydrolase activity was measured in serial 1:2 dilutions of plasma for 30 minutes at 25°C in a Tris buffer (0.15 M Tris, 6% BSA, 17.9 U/mL heparin, pH 8.5) with a final NaCl concentration of 0.13 M. Total TG hydrolase activity was calculated from dilutions falling within the linear range of the dilution curve; 1 mU of TG hydrolase activity corresponds to 1 nmol fatty acid release per minute. TG hydrolase activity resulting from the activity of mouse LPL was determined according to procedures validated by Dallinga-Thie and coworkers (42).

LPL mass was measured with a sandwich ELISA. Wells of 96-well plates (Costar) were coated with a rabbit IgG against mouse LPL (no. 3175; 100 μL/well, 0.5 μg/well) overnight at 4°C. After washing wells with PBS with Ca^2+^/Mg^2+^ and 5 U/mL heparin and 0.1% BSA (the same buffer was used in all washing steps), the plates were blocked for 4 hours at room temperature with StartingBlock buffer (200 μL/well, Thermo Fisher Scientific). Serial 1:2 dilutions of recombinant mouse LPL (0–50 ng/mL) were used for the standard curve. Serial dilutions of plasma samples were added to the plate and incubated overnight at 4°C. All serial dilutions were performed in StartingBlock buffer containing 10 U/mL heparin. After washing the plates, a rat monoclonal antibody against mouse LPL (27A; 100 μL/well, 100 ng/well) was added to the wells and incubated for 2 hours at room temperature. Binding of the monoclonal antibody to the captured LPL was detected with an HRP-labeled donkey anti–rat IgG (Jackson ImmunoResearch). After washing, 1-step Ultra TMB substrate (50 μL/well, Thermo Fisher Scientific) was added to the wells for 2 minutes at room temperature. The reaction was stopped with 50 μL of 2 M sulfuric acid, and the optical density (OD) was read at 450 nm on a SpectraMax iD3 plate reader (Molecular Devices). The amount of LPL in plasma samples was calculated by linear regression from dilutions that fell within the linear range of the standard curve.

### Kinetics of mouse LPL–mouse GPIHBP1 interactions by surface plasmon resonance.

The reaction kinetics between purified mouse LPL and mouse GPIHBP1 proteins were measured with a Biacore T200 instrument using a modified version of a protocol optimized for bovine LPL (8). LPL was captured on the sensor chip with the mouse LPL–specific antibody 27A, which binds to the tryptophan-rich lipid-binding loop in LPL’s C-terminal domain. Antibody 27A was immobilized on the chip with amine chemistry, and excess binding sites were blocked with 1 M ethylenediamine. Purified mouse LPL was captured on the chips by injecting 100 nM mouse LPL at 20 μL/min for 200 seconds in 10 mM HEPES (pH 7.4), 300 mM NaCl, 4 mM CaCl_2_, 10% (vol/vol) glycerol, 0.05% (vol/vol) surfactant P20, 1 mg/mL fatty acid–free BSA, 0.1 mg/mL carboxymethyl dextran, and 0.05% (wt/vol) NaN_3_. This protocol minimized unfolding of LPL and resulted in capture densities of 400 resonance units (RUs) (8 fmol LPL/mm^2^). To minimize run time and LPL unfolding, single-cycle kinetics were used to measure GPIHBP1-LPL binding, and we used only 3 cycles per LPL capture (2 buffer blanks and 1 sample). The running buffer was 10 mM HEPES (pH 7.4), 150 mM NaCl, 4 mM CaCl_2_, 0.05% (vol/vol) P20, 0.2 mg/mL fatty acid–free BSA, and 0.05% (wt/vol) NaN_3_. We performed identical studies at a higher concentration of NaCl (750 mM). Our protocols included 5 consecutive injections of 2-fold dilutions of WT-GPIHBP1 (0.25–4 nM) and S-GPIHBP1 (2–32 nM) at a flow rate of 50 μL/min at 20°C. At the end of the third cycle, two 10-μL injections of 10 mM glycine HCl (pH 1.5) were used to regenerate the chip. The sensorgrams were double-buffer referenced; the binding rate constants were calculated by fitting the data to a bimolecular interaction model with the mathematical model developed for single-cycle kinetics (T200 Evaluation Software 3.0, GE Healthcare).

### CHO cell transfection studies.

LPL and GPIHBP1 were expressed with CMV promoter–based vectors (1, 13, 43) in CHO cells that had been modified by CRISPR/Cas9 editing to eliminate expression of hamster LPL (44). A vector for S-GPIHBP1 was generated by replacing sequences for GPIHBP1 residues 32 to 48 with sequences for the S-protein tag. The integrity of plasmids was verified by DNA sequencing. Transient transfections were carried out by electroporating 2 to 5 μg of plasmid DNA into 1 × 10^6^ to 5 × 10^6^ cells with the Nucleofector II apparatus (Lonza) and the Cell line T Nucleofector kit (Lonza). To assess whether the GPIHBP1 on the cell surface was GPI anchored, cells were incubated for 20 minutes at 37°C in medium containing 10 U/mL phosphatidylinositol-specific phospholipase C (PIPLC) (Thermo Fisher Scientific). Samples of the medium and cell lysates were collected for Western blot analyses.

Immunofluorescence microscopy was used to test the ability of S-GPIHBP1 and W108S-GPIHBP1 to capture LPL from the cell culture medium (13). (The W108S substitution, located in the LU domain, prevents LPL binding; refs. 5, 12). Cells that had been transfected with S-GPIHBP1 or W108S-GPIHBP1 were coplated on coverslips in 24-well plates with cells that had been transfected with V5-tagged mouse LPL. On the following day, the cells were cooled on ice for 15 minutes, washed 6 times with ice-cold PBS/Ca^2+^/Mg^2+^, and blocked for 1 hour at 4°C in 10% donkey serum. The cells were then incubated overnight at 4°C in 3% donkey serum containing antibody 3175 (10 μg/mL) and 11A12 (5 μg/mL). After washing, the cells were fixed in 4% paraformaldehyde (PFA), permeabilized with 0.2% Triton X-100, and blocked in 10% donkey serum for 1 hour at room temperature. Cells were then incubated with a mouse monoclonal antibody against the V5 tag (10 μg/mL) for 1 hour at room temperature, followed by a 30-minute incubation at room temperature with an Alexa Fluor 647–conjugated donkey anti–rabbit IgG, an Alexa Fluor 568–conjugated donkey anti–mouse IgG, and an Alexa Fluor 488–conjugated donkey anti–rat IgG (Thermo Fisher Scientific; all at 2.5 μg/mL). The coverslips were mounted on glass slides with ProLong Diamond antifade mounting media containing DAPI (Thermo Fisher Scientific). Confocal images, recorded on an LSM980 microscope (Zeiss) with a 20× objective and 3.5× digital zoom, were processed with Zen Blue software (Zeiss).

### Detecting binding of an S protein–specific antibody to the capillary lumen.

Anesthetized mice were perfused with 10 mL of Tyrode’s solution (136 mM NaCl, 5.4 mM KCl, 0.33 mM NaH_2_PO_4_, 1 mM MgCl_2_, 10 mM HEPES [pH 7.4], 10 mM glucose) through the inferior vena cava. Hearts from *Gpihbp1*^S/+^ and *Gpihbp1*^+/+^ mice were removed, and the aorta cannulated with a blunt-end 20-gauge needle and secured with a suture. Hearts were flushed with Tyrode’s solution, submerged in 30 mL of Tyrode’s solution, and perfused with a 1-mL solution containing 50 μg/mL Alexa Fluor 488–labeled 11A12, 50 μg/mL Alexa Fluor 555–labeled antibody against the S-protein tag, and 50 μg/mL Alexa Fluor 647–labeled rat IgG (as a perfusion control). After 5 minutes, hearts were perfused with 10 mL Tyrode’s solution followed by 5 mL of 3% PFA in PBS. The hearts were frozen in OCT and processed for fluorescence microscopy.

### Confocal microscopy.

To detect GPIHBP1 and LPL in mouse tissues, 10-μm-thick frozen sections were prepared, fixed with 3% PFA, permeabilized with 0.2% Triton X-100 in PBS for 5 minutes, and then incubated in blocking buffer containing 0.2% BSA and 5% donkey serum in PBS for 1 hour at room temperature. Sections were incubated with primary antibodies at 4°C overnight (5 μg/mL antibody 11A12; 20 μg/mL 2H8; 4 μg/mL goat anti-CD31 polyclonal antibody, 10 μg/mL 3174 or 3175, 10 μg/mL goat anti-LPL antibody). Sections were washed 3 times to remove unbound antibody and then incubated with fluorescently labeled secondary antibodies for 1 hour at room temperature. Secondary antibodies were DyLight 650–labeled anti–rat IgG (Thermo Fisher Scientific), Alexa Fluor 549–labeled anti–hamster IgG (Jackson ImmunoResearch), Alexa Fluor 488–labeled anti–rabbit IgG (Thermo Fisher Scientific), and Alexa Fluor 568–labeled anti–goat IgG (Thermo Fisher Scientific), all at a 1:200 dilution. After washing 3 times, the sections were postfixed with 3% PFA, and DNA was stained with DAPI. Images were obtained with an LSM980 microscope (Zeiss) with 20× or 63× objectives. The intensities of LPL and CD31 fluorescence signals on capillary ECs in low-magnification images were quantified with ImageJ (NIH). The intensities of the LPL, CD31, and GPIHBP1 fluorescence signals in high-magnification capillary cross sections were quantified with Zen Blue software (Zeiss). GPIHBP1/CD31 and LPL/CD31 fluorescence intensity ratios in the APM and LPM were normalized to the ratio in the “combined APM/LPM segment,” where the APM and LPM were not separated by a cell nucleus.

### Assessing intravascular binding of antibodies conjugated to infrared dyes.

Mice were injected intravenously with 100 μg of IRDye 800–11A12 (or IRDye 800–3174) and 100 μg of IRDye 680–2H8 in a volume of 0.2 mL. After 3 minutes, mice were perfused with 10 mL PBS (2–3 mL/min) through the left ventricle, followed by perfusion with 10 mL of 3% PFA. The tissues were embedded in OCT, 10-μm-thick frozen sections (10/mouse) were prepared, and sections were scanned with an Odyssey infrared scanner (LI-COR). The signal intensity for antibody 11A12 or 3174 was normalized to the 2H8 signal. Tissue area was quantified with ImageStudio software (LI-COR).

### Testing the abilities of WT-GPIHBP1 and S-GPIHBP1 to extract mouse LPL from an HSPG-coated sensor chip.

Streptavidin-coupled CM5 sensor chips were coated with biotinylated heparin DP4 fragments. The active surface had a highly sulfated version of that fragment (M08S09b), with sulfate modifications on the N-, O-2, and O-6 groups (Iduron); a nonbinding reference surface had a nonsulfated fragment (M08 S00). The biotin was added by click coupling chemistry between a *para*-(6-azido hexanamido)phenyl tag at the reducing end of the heparin fragment and PEG4-biotin with an alkyne group. Only the sulfated oligosaccharide bound LPL, and those interactions were transient. Very high surface densities of the heparin fragment on the sensor chip (300 fmol/mm^2^) were used to promote electrostatic binding of LPL with pronounced mass transport limitation. For each cycle, 25 nM mouse LPL was loaded for 200 seconds at a flow rate of 20 μL/min in 10 mM HEPES (pH 7.4), 150 mM NaCl, 4 mM CaCl_2_, 10% (vol/vol) glycerol, 0.05% (vol/vol) surfactant P20, 1 mg/mL fatty acid–free BSA, 1 μM GPIHBP1^1–33^ (an AD peptide; residues 1–33 of human GPIHBP1), and 0.05% (wt/vol) NaN_3_. The low concentration of the human AD peptide in the buffer did not interfere with the binding of LPL to the high surface density of heparin fragments and was included to stabilize the LPL and promote uniform LPL capture on the sensor chip. This procedure resulted in LPL capture levels of 13 fmol/mm^2^. The ability of GPIHBP1 to bind and extract LPL from the dynamic reservoir was tested by injecting five 2-fold serial dilutions of 12.5 to 200 nM WT-GPIHBP1 or S-GPIHBP1, 5 times for 250 seconds each at 20 μL/min, in 10 mM HEPES (pH 7.4), 150 mM NaCl, 4 mM CaCl_2_, 10% (vol/vol) glycerol, 0.05% (vol/vol) surfactant P20, 0.2 mg/mL fatty acid–free BSA, and 0.05% (wt/vol) NaN_3_. At the end of each cycle, 2 consecutive injections of 10 μL 3 M guanidinium chloride regenerated the chip.

### Visualizing movement of GPIHBP1 across capillary endothelial cells in living mice.

To examine GPIHBP1 transport across ECs, a BAT pad of *Gpihbp1^+/+^* and *Gpihbp1*^S/S^ mice was injected (using a 29-gauge needle) with 15 μL of normal saline containing 3 μg of Alexa Fluor 488–11A12 and 3 μL of India Ink and/or a fluorescently labeled tomato lectin (to visualize the injected area). In some studies, Alexa Fluor 488–11A12 was coinjected with (a) 0.75 U heparin (McKesson) and 15 μg dextran sulfate (Calbiochem); (b) 34 μmol of a synthetic peptide corresponding to the WT GPIHBP1 AD (EDGDADPEPENYNYDDDDDEEEEEE); or (c) 34 μmol of a synthetic peptide corresponding to the S-protein tag (KETAAAKFERQHMDS). After 15 or 90 minutes, mice were perfused with PBS and perfusion fixed with 3% PFA. Frozen sections were prepared, and DNA was stained with DAPI. Confocal micrographs of capillary cross sections (*n* = 50/group) were used to assess the efficiency of Alexa Fluor 488–11A12 transport to the LPM.

### Thermal stability of mouse LPL by differential scanning fluorimetry.

The thermal stability of purified mouse LPL was assessed by differential scanning fluorimetry with a Prometheus NT.48 instrument (Nanotemper). Mouse LPL (6 μM), either alone or with an equimolar amount of WT-GPIHBP1 or S-GPIHBP1, was prepared in 10 mM HEPES, 150 mM NaCl (pH 7.4) and incubated on ice for 3 minutes to permit complex formation while limiting LPL unfolding (21). All samples were heated with a ramping temperature of 1°C/min from 20°C to 95°C with continuous excitation of tryptophans and measurement of fluorescence emission at 330 nm. All samples were measured in triplicate. The apparent melting temperature (*T_m_*) was determined with PR.Stability software (Nanotemper) using a 2-step fit of the first derivative of the 330 nm fluorescence in the temperature range from 27°C to 81°C.

### Measuring the impact of GPIHBP1 on the stability of LPL TG lipase activity.

To assess LPL stability, we incubated mouse LPL (18.87 μM) alone or with mouse WT-GPIHBP1 or S-GPIHBP1 (GPIHBP1/LPL molar ratio of 5:1). All incubations were carried out at 25°C in 20 mM Tris (pH 8.0), 150 mM NaCl, and 0.2% fatty acid–free BSA. TG hydrolase activities at different time points were quantified with a [^3^H]triolein substrate (19, 45).

### Statistics.

GraphPad Prism 9.0 was used for statistical analyses. Bar graphs show mean ± SEM. A 2-tailed Student’s *t* test was used for comparisons between 2 independent groups. For multiple group comparisons, 1- or 2-way ANOVA tests were used.

### Study approval.

All studies were approved by UCLA’s Animal Research Committee according to guidelines described in the NIH *Guide for the Care and Use of Laboratory Animals* (National Academies Press, 2011).

## Author contributions

SGY, WS, LGF, APB, and MP designed the experiments and wrote and formatted the manuscript. WS, LGF, APB, MP, AMLW, KKK, ALG, YY, YT, PM, JM, H Jung, PJJ, CJJ, and KM performed, collected, and assembled the experiments. WS, SGY, LGF, APB, MP, and PT secured funding. WS, APB, MP, AMLW, KKK, ALG, KM, TK, KN, MM, GB, and H Jiang assisted with resources and/or provided expertise and feedback.

## Supplementary Material

Supplemental data

## Version 1 03/01/2022

Electronic publication
