# Supplementary material for: Electrostatic sheathing of lipoprotein lipase is essential for its movement across capillary endothelial cells
Source: J Clin Invest. 2022 Mar 1;132(5):e157500. doi: 10.1172/JCI157500 (PMC8884915; doi:10.1172/JCI157500)

Supplemental data for:

Electrostatic sheathing of lipoprotein lipase is essential for its movement across capillary endothelial cells

Wenxin Song[1], Anne P. Beigneux[1], Anne-Marie Lund Winther[2,3], Kristian Kølby Kristensen[2,3], Anne Louise Grønnemose[2,3], Ye Yang[1], Yiping Tu[1], Priscilla Munguia[1], Jazmin Morales[1], Hyesoo Jung[1], Pieter J. de Jong[4], Cris J. Jung[4], Kazuya Miyashita[5,6], Takao Kimura[5], Katsuyuki Nakajima[5], Masami Murakami[5], Gabriel Birrane[7], Haibo Jiang[8], Peter Tontonoz[9], Michael Ploug[2,3,†], Loren G. Fong[1,†], and Stephen G. Young[1,10,†]

From the [1]Department of Medicine, David Geffen School of Medicine, University of California, Los Angeles, CA 90095; [2]Finsen Laboratory, Rigshospitalet, DK–2200 Copenhagen N, Denmark; [3]Biotech Research and Innovation Centre (BRIC), University of Copenhagen, DK-220 Copenhagen N, Denmark; [4]Children's Hospital Oakland Research Institute, Oakland, CA 94609; [5]Department of Clinical Laboratory Medicine, Gunma University, Graduate School of Medicine, Maebashi, Gunma, Japan; [6]Immuno-Biological Laboratories (IBL), Fujioka, Gunma, Japan; [7]Division of Experimental Medicine, Beth Israel Deaconess Medical Center, Boston, Massachusetts 02215; [8]Department of Chemistry, The University of Hong Kong, Hong Kong; [9]Department of Pathology and Laboratory Medicine, University of California, Los Angeles, CA 90095; [10]Department of Human Genetics, David Geffen School of Medicine, University of California, Los Angeles, CA 90095.

Supplemental Figures and Figure Legends

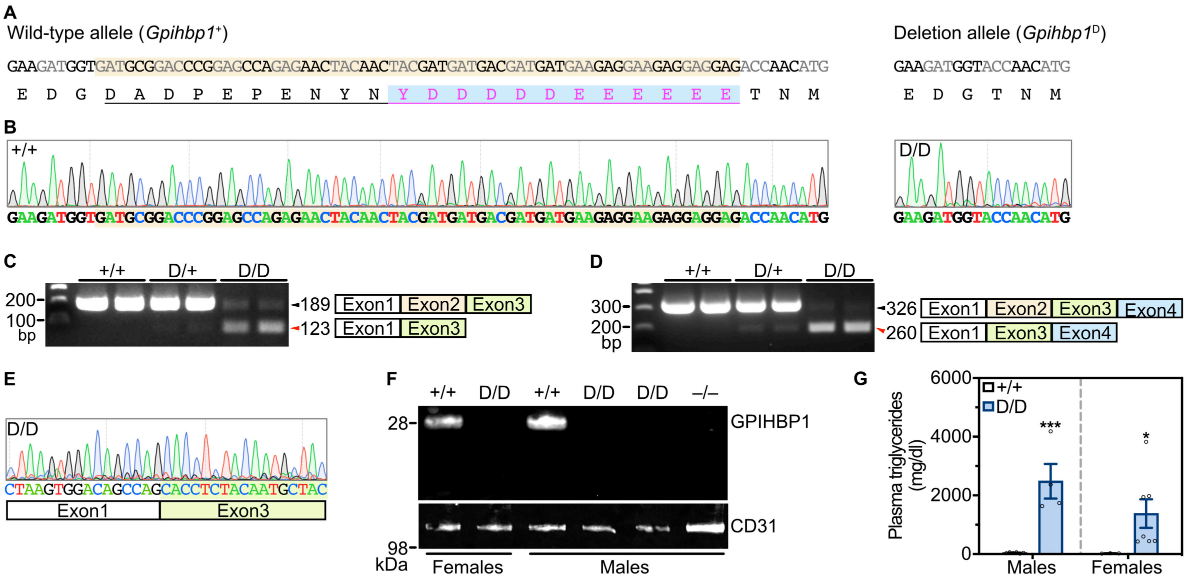

Supplemental figure 1. A mutant *Gpihbp1* allele with a deletion of GPIHBP1's acidic domain. (**A**) Nucleotide and amino acid sequences for the wild-type (WT) allele (*Gpihbp1*$^{+}$) and a "deletion allele" (*Gpihbp1*^{D}). In *Gpihbp1*^{D}, 66 bp in exon 2 (highlighted *salmon* in the *Gpihbp1*$^{+}$ allele) were removed, thereby deleting 22 amino acids (*underlined*), including the sulfated tyrosine and the long stretch of acidic residues (DDDDDEEEEEE) (*magenta*). (**B**) Sequencing chromatograms from *Gpihbp1*$^{+/+}$ and *Gpihbp1*$^{D/D}$ mice (deleted nucleotides highlighted *salmon* in the *Gpihbp1*$^{+}$ sequence). Sequencing of the *Gpihbp1*^{D} allele revealed no other mutations. (**C**) PCR products from heart cDNA of *Gpihbp1*$^{+/+}$*, Gpihbp1*$^{D/+}$*,* and *Gpihbp1*$^{D/D}$ mice (forward primer in exon 1; reverse primer in exon 3). A 189-bp fragment (black arrowhead) was amplified in *Gpihbp1*$^{+/+}$ mice; a faint 189-bp band was amplified in *Gpihbp1*$^{D/D}$ mice, but the main product was 123 bp (*red* arrowhead, reflecting exon 2 skipping). (**D**) PCR products from heart cDNA from *Gpihbp1*$^{+/+}$*, Gpihbp1*$^{D/+}$, and *Gpihbp1*$^{D/D}$ mice (forward primer in exon 1; reverse primer in exon 4). A 326-bp fragment (black arrowhead) was amplified from the *Gpihbp1*$^{+}$ allele; a faint 326-bp product was amplified from the *Gpihbp1*^{D} allele, but the main product was 260 bp (*red* arrowhead, reflecting exon 2 skipping). (**E**) Sequencing of the 260-bp product from heart cDNA of *Gpihbp1*$^{D/D}$ mice (Figure ID in the Data Supplement), revealing exon 2 skipping. (**F**) Western blots of heart extracts from *Gpihbp1*$^{+/+}$, *Gpihbp1*$^{D/D}$, and *Gpihbp1*$^{-/-}$ mice with the GPIHBP1-specific antibody (11A12) and a CD31 antibody. The 11A12 epitope, located between GPIHBP1's LU domain and its carboxyl-terminal signal peptide, is encoded by exon 4. (**G**) Plasma triglyceride levels in 10-week-old mice ($n = 6$ male *Gpihbp1*$^{+/+}$; $n = 4$ male *Gpihbp1*$^{D/D}$; $n = 3$ female *Gpihbp1*$^{+/+}$; $n = 7$ female *Gpihbp1*$^{D/D}$ mice). Data are represented as mean ± SEM. *$p < 0.05$; ***$p < 0.001$. A two-tailed Student's t test was used to compare the means in panel **G**.

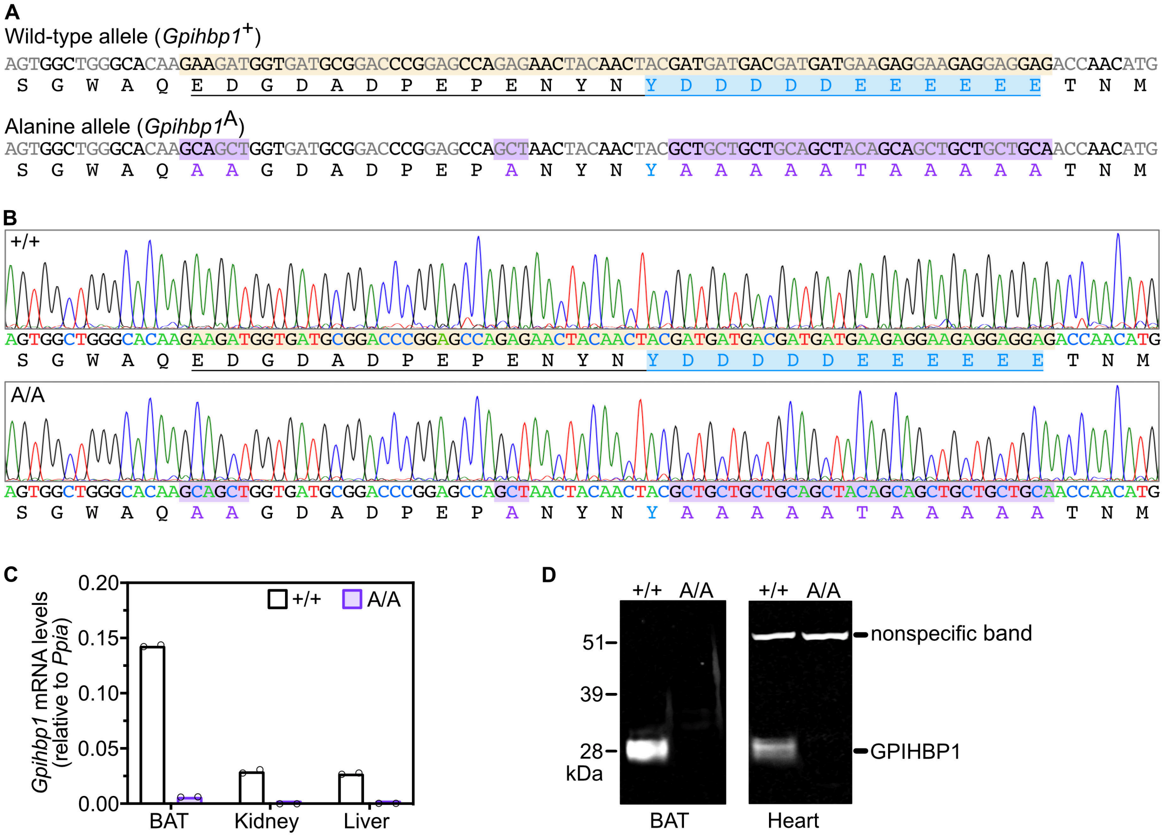

Supplemental figure 2. A mutant *Gpihbp1* allele in which most of the acidic residues in the acidic domain were replaced with alanine. (**A**) Nucleotide and amino acid sequences of a wild-type allele (*Gpihbp1*$^{+}$) and an alanine-substituted allele (*Gpihbp1*^{A}). In the *Gpihbp1*$^{+}$ allele, the stretch of nucleotides that was altered in the *Gpihbp1*^{A} allele is highlighted *salmon*; the stretch of amino acids that was changed in the *Gpihbp1*^{A} allele is *underlined*. The sulfated tyrosine and long stretch of acidic residues in the *Gpihbp1*$^{+}$ allele are highlighted *blue*. The mutated codons in the *Gpihbp1*^{A} allele are highlighted *purple*; the altered amino acids are *purple*. (**B**) Sequencing chromatograms from *Gpihbp1*$^{+/+}$ and *Gpihbp1*$^{A/A}$ mice. Sequencing of the *Gpihbp1*^{A} allele revealed no other mutations. (**C**) *Gpihbp1* transcript levels in *Gpihbp1*$^{+/+}$ and *Gpihbp1*$^{A/A}$ mice, as judged by qRT-PCR. *Gpihbp1* transcript levels in the brown adipose tissue (BAT), kidney, and liver of *Gpihbp1*$^{A/A}$ mice (n = 2/group) were reduced by 95.7%, 98.8%, and 97.7%, respectively. (**D**) Western blots of BAT and heart extracts from *Gpihbp1*$^{+/+}$ and *Gpihbp1*$^{A/A}$ mice with the GPIHBP1-specific antibody 11A12. GPIHBP1 was detected in *Gpihbp1*$^{+/+}$ tissue extracts but not *Gpihbp1*$^{A/A}$ extracts. A ~50-kDa nonspecific band was observed in heart extracts. Consistent with the absence of GPIHBP1, the plasma of chow-fed *Gpihbp1*$^{A/A}$ mice was lipemic, with plasma triglyceride levels >1,500 mg/dl.

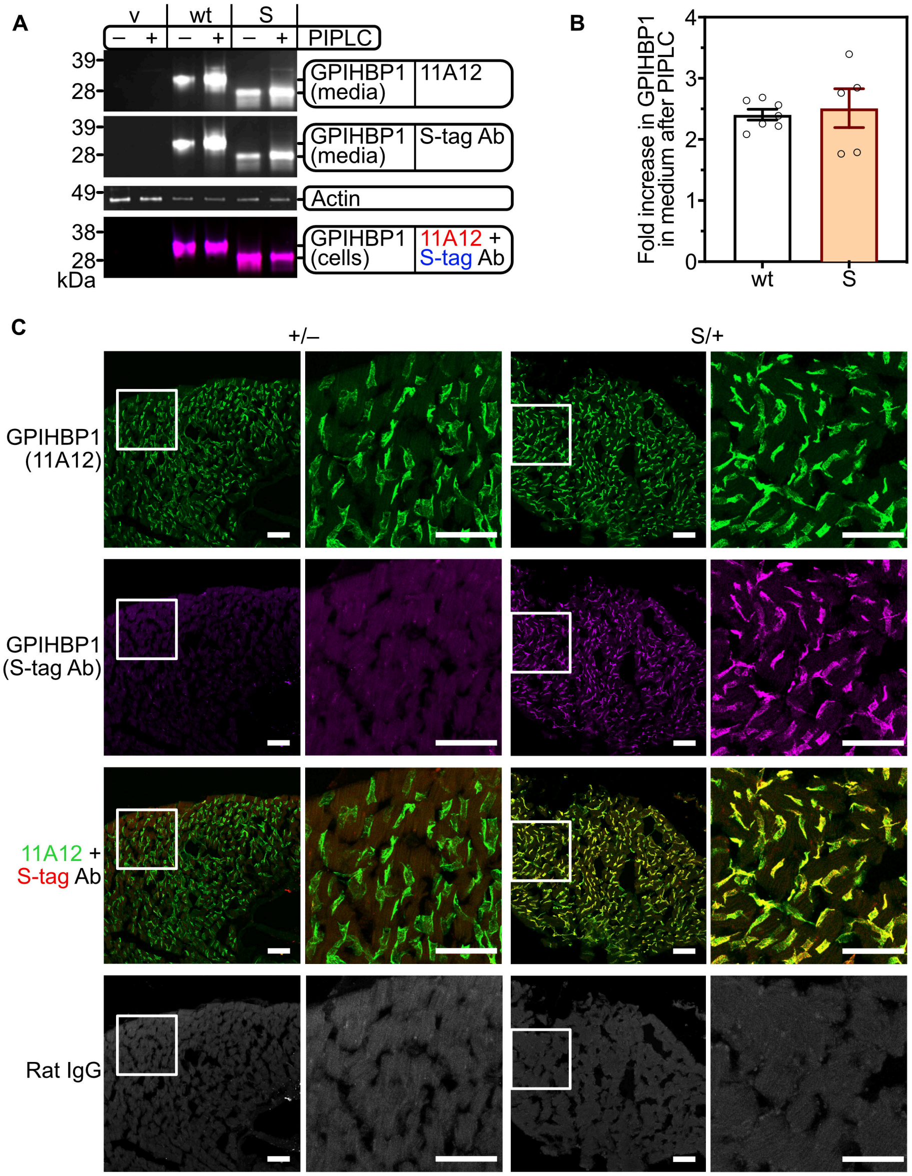

Supplemental figure 3. S-GPIHBP1 is expressed on the surface of transfected CHO cells and is expressed on the luminal surface of heart capillary endothelial cells. (**A**) Western blots of GPIHBP1 in the medium and cell extracts of CHO cells that had been transfected with an empty expression vector (v), a vector for WT-GPIHBP1 (wt) that contained an N-terminal S-protein tag, or a vector encoding S-GPIHBP1. One day after the transfection, cells were incubated for 10 min in the absence (–) or presence (+) of phosphatidylinositol-specific phospholipase C (PIPLC, 10 U/ml). Western blots were performed with the GPIHBP1-specific antibody 11A12 and an S-protein (S-tag) antibody. (**B**) Fold increase in GPIHBP1 in the cell culture medium after incubating WT-GPIHBP1– and S-GPIHBP1–transfected cells with PIPLC (n = 3 western blot experiments). Data are represented as mean ± SEM. (**C**) Confocal micrographs of heart sections from *Gpihbp1*$^{+/–}$ and *Gpihbp1*$^{S/+}$ mice after perfusing cannulated hearts with fluorescently labeled antibodies [the

GPIHBP1-specific antibody 11A12 (*green*), an antibody against the S-protein tag (second row, *magenta*; third row, *red*), and nonimmune rat IgG (*white*)]. Boxed images are shown at a higher magnification to the right. Scale bar, 50 µm. A two-tailed Student's *t* test was used to compare means in panel **B**.

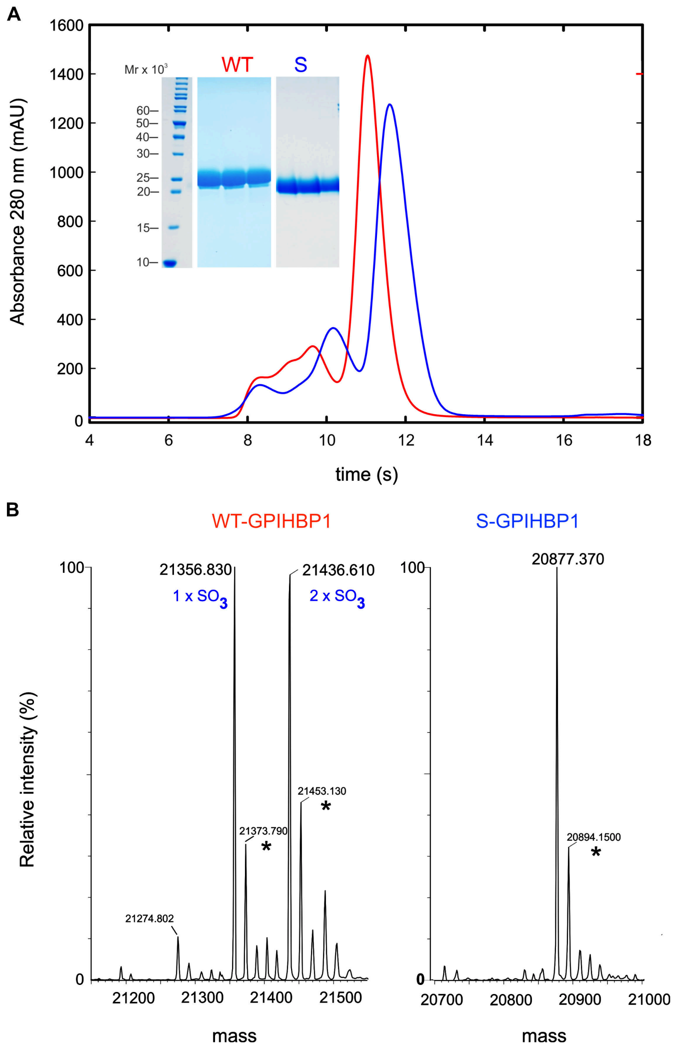

Supplemental figure 4. Characterization of purified preparations of WT-GPIHBP1 and S-GPIHBP1. (**A**) WT-GPIHBP1 (*red*) and S-GPIHBP1 (*blue*) were analyzed by size-exclusion chromatography (SEC) with a Sepharose G75 column. The position and Gaussian shape of the elution profiles for the major peaks revealed that these preparations contained primarily monomeric GPIHBP1. Three peak fractions were analyzed with Coomassie blue–stained SDS-polyacrylamide gels (inset). (**B**) Mass spectrometry of WT-GPIHBP1 and S-GPIHBP1 proteins revealed a biantennary N-linked glycan (1039 Da). As shown for human GPIHBP1 (Kristensen et al., 2018), the N-terminal glutamine residue is prone to deamination (conversion to a pyroglutamate). The acidic domain of WT-GPIHBP1 contains two tyrosines (Tyr^{35} and Tyr^{37}); neither is present in S-GPIHBP1. The mass spectrum for purified WT-GPIHBP1 preparation revealed that ~one-half of the WT-GPIHBP1 molecules contained two sulfated tyrosines, while the other half contained one sulfated tyrosine.

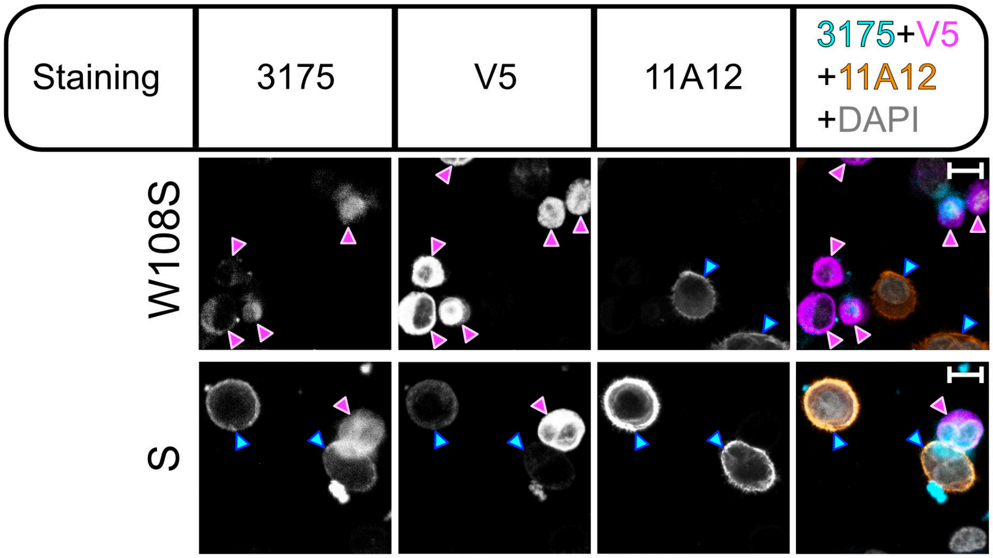

Supplemental figure 5. S-GPIHBP1 binds mouse LPL whereas W108S-GPIHBP1 does not. The W108S substitution disrupts the hydrophobic GPIHBP1–LPL binding interface (Birrane et al., 2019). CHO cells were transfected with expression vectors for S-GPIHBP1 (S) or W108S-GPIHBP1 and then plated on coverslips with either mock-transfected cells or cells that had been transfected with V5-tagged mouse LPL. On the following day, the cells were incubated at 4°C with a mouse LPL–specific 3175 IgG and the GPIHBP1-specific antibody 11A12. After washing, the cells were fixed and permeabilized. Next, the cells were incubated with a V5-specific antibody, making it possible to identify cells that expressed mouse LPL (*magenta* arrowhead). 3175 detected LPL binding to the surface of S-GPIHBP1-expressing cells (*blue* arrowhead). The V5 antibody also detected S-GPIHBP1-bound LPL on the cell surface. There was no binding of 3175 or V5 antibodies to the surface of cells that expressed W108S-GPIHBP1. In the merged image (last panel on the *right*), 3175 is *blue* (Alexa Fluor 647); the V5 antibody is *magenta* (Alexa Fluor 568); 11A12 is *orange* (Alexa Fluor 488). DNA was stained with DAPI (*white*). Scale bar, 10 µm.

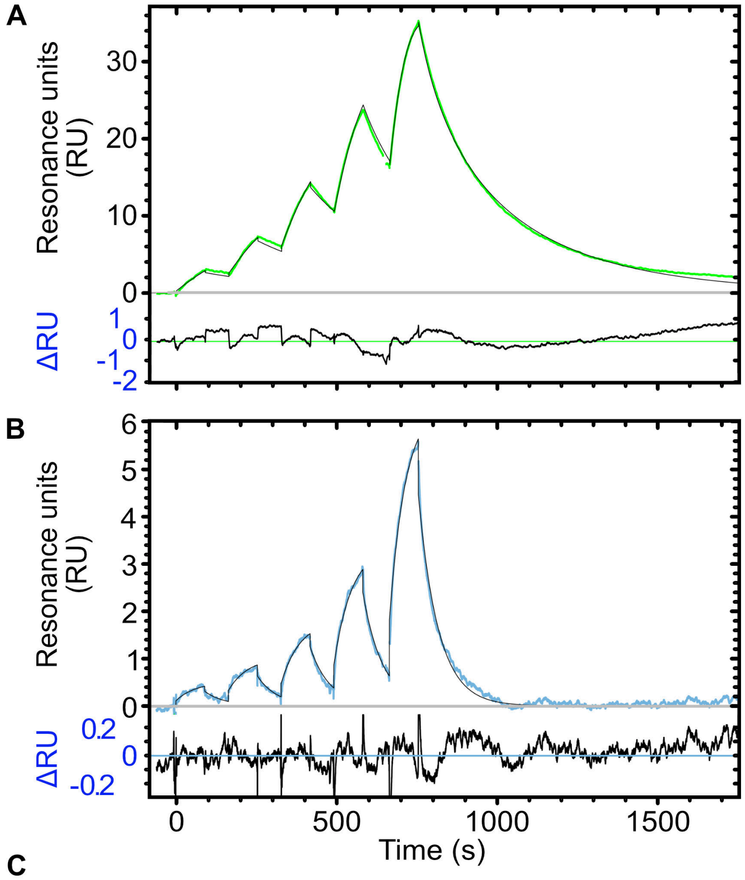

C

	k_{on} (10^6 $M^{-1}s^{-1}$)	k_{off} (10^{-2} s^{-1})	K_D (nM)
wt (150 mM NaCl)	13.5 ± 0.18	1.25 ± 0.02	0.93
S (150 mM NaCl)	0.16 ± 0.04	1.67 ± 0.33	107
wt (750 mM NaCl)	0.70 ± 0.002	1.43 ± 0.002	20
S (750 mM NaCl)	0.27 ± 0.006	4.95 ± 0.035	183
S to DP4-bound LPL	0.07 ± 0.0005	2.11 ± 0.01	290

Supplemental figure 6. Binding kinetics for interactions between mouse LPL and either WT-GPIHBP1 or S-GPIHBP1. (**A–B**) Sensorgrams (at 150 mM NaCl) for the binding of WT-GPIHBP1 (**A**) or S-GPIHBP1 (**B**) to mouse LPL (captured on the sensor chip with the mouse LPL–specific monoclonal antibody 27A). Single-cycle analyses involved five consecutive injections of twofold dilutions of WT-GPIHBP1 (0.25–4 nM) and S-GPIHBP1 (2–32 nM). (**C**) Table summarizing the kinetic rate constants (mean ± SD) in studies performed at 150 mM or 750 mM NaCl. Rate constants were derived from the global fitting of SPR data to a simple bimolecular interaction model. The kinetic data for binding of S-GPIHBP1 to heparin (DP4)-bound LPL was generated from the experiment shown in Figure 6 (performed in the presence of 150 mM NaCl).

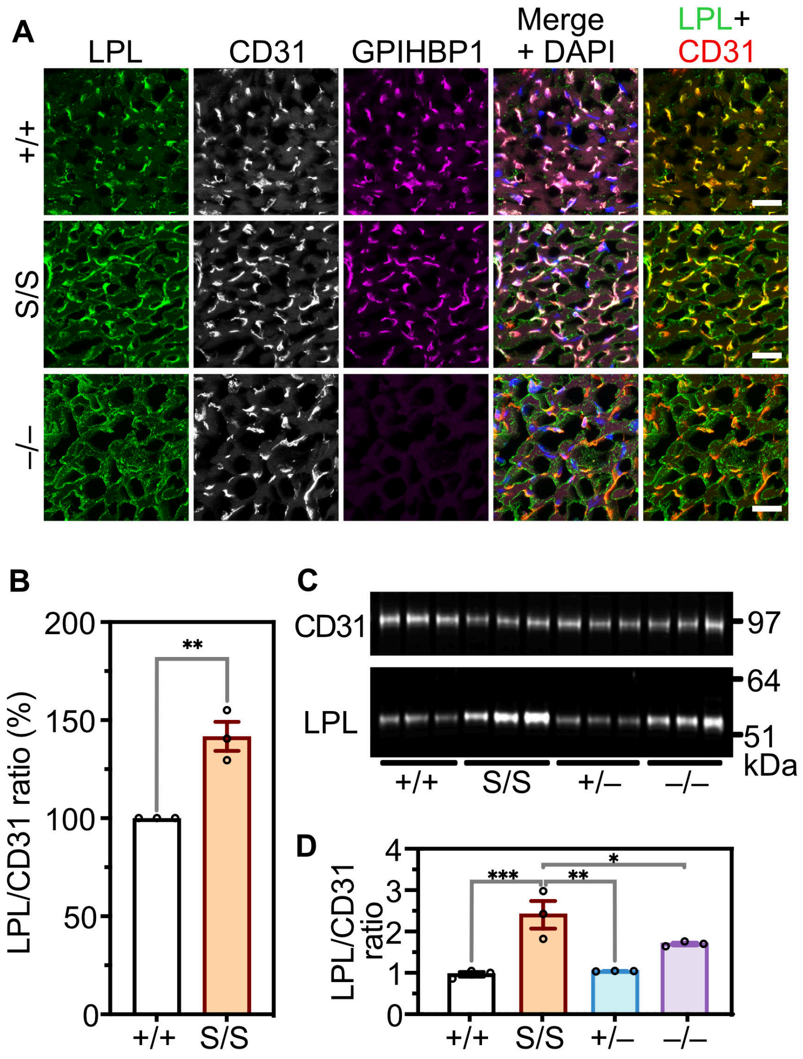

Supplemental figure 7. LPL, CD31, and GPIHBP1 expression in heart from *Gpihbp1*$^{+/+}$, *Gpihbp1*$^{S/S}$, *Gpihbp1*$^{+/–}$, and *Gpihbp1*$^{–/–}$ mice. (**A**) Confocal immunofluorescence studies of LPL, CD31, and GPIHBP1 expression in hearts from *Gpihbp1*$^{+/+}$, *Gpihbp1*$^{S/S}$, and *Gpihbp1*$^{–/–}$ mice. Scale bar, 20 µm. (**B**) LPL/CD31 fluorescence intensity ratios in heart capillaries of *Gpihbp1*$^{+/+}$ and *Gpihbp1*$^{S/S}$ mice. Fluorescent intensity ratios were quantified in three independent experiments (>100 capillaries/genotype). The ratio in *Gpihbp1*$^{S/S}$ capillaries in each experiment was normalized to the ratio in *Gpihbp1*$^{+/+}$ capillaries (set at 1.0). Data are represented as mean ± SEM. $^{**}p < 0.01$. (**C**) Western blot studies of CD31 and LPL levels in heart extracts from *Gpihbp1*$^{+/+}$, *Gpihbp1*$^{S/S}$, *Gpihbp1*$^{+/–}$, and *Gpihbp1*$^{–/–}$ mice (n = 3/group). Each lane represents an individual mouse. (**D**) LPL/CD31 ratios in heart extracts, as judged by quantification of western blot band intensities in panel C. The LPL/CD31 band intensity ratio in *Gpihbp1*$^{S/S}$ mice was higher than in the other groups of mice. Data are represented as mean ± SEM. $^{*}p < 0.05$; $^{**}p < 0.01$; $^{***}p < 0.001$. Means were compared with a two-tailed Student's t test in panel **B**, and with a one-way ANOVA test in panel **D**.

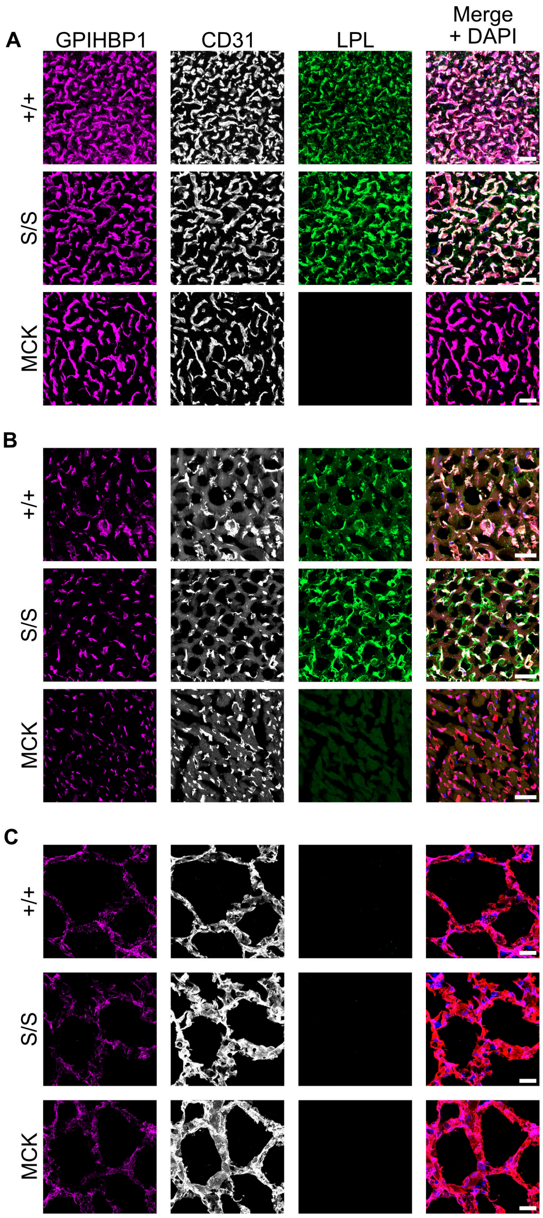

Supplemental figure 8. Immunofluorescence microscopy to assess GPIHBP1, CD31, and LPL expression in brown adipose tissue (BAT) (A), heart (B), and lung (C) of *Gpihbp1*$^{+/+}$, *Gpihbp1*$^{S/S}$, and "MCK mice." MCK mice are *Lpl*$^{-/-}$ mice with a human LPL transgene driven by the promoter of the muscle creatine kinase gene. Images were recorded with the same microscope settings. Scale bar, 20 µm.

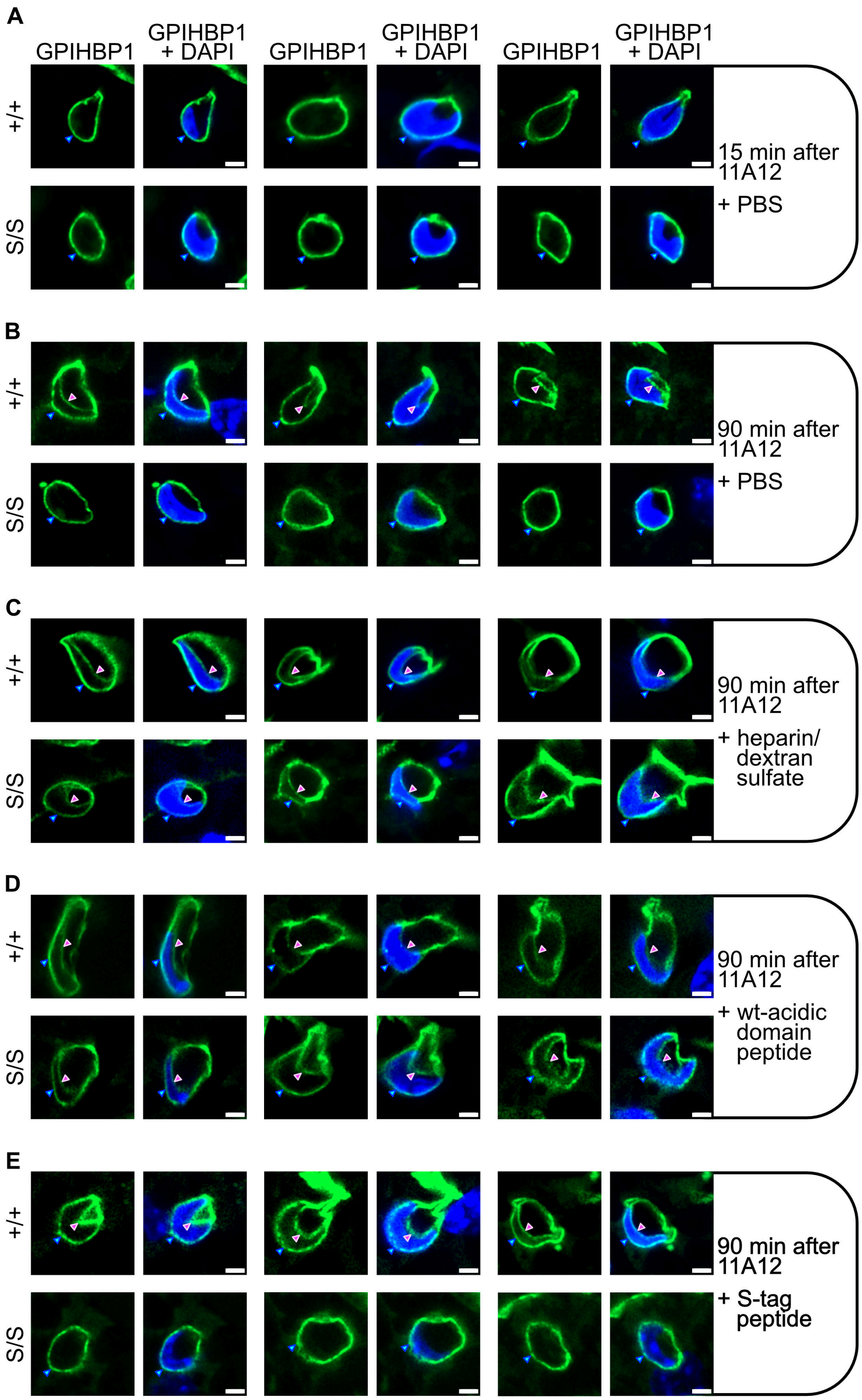

Supplemental figure 9. Assessing movement of the GPIHBP1-specific antibody 11A12 from

the abluminal plasma membrane (APM) to the luminal plasma membrane (LPM) in brown adipose tissue (BAT) capillary endothelial cells of living mice. Alexa Fluor 488–11A12 (*green*) was injected into the interscapular BAT of *Gpihbp1*$^{+/+}$ and *Gpihbp1*$^{S/S}$ mice. After 15 min or 90 min, mice were perfused with PBS; perfusion-fixed with PFA; and frozen sections were prepared. Images of capillary cross sections containing an endothelial cell nucleus were recorded by fluorescence microscopy. The presence of the cell nucleus made it possible to visualize 11A12 at the APM (*blue* arrowhead) and the LPM (*magenta* arrowhead). DNA was stained with DAPI (blue). Shown here are three capillary cross sections/experimental condition. Scale bars, 2 µm. An additional capillary cross section for each experimental condition is shown in Fig. 8. (**A**) Capillary cross sections in BAT from *Gpihbp1*$^{+/+}$ and *Gpihbp1*$^{S/S}$ mice 15 min after the injection of Alexa Fluor 488–11A12. (**B**) Capillary cross sections in BAT from *Gpihbp1*$^{+/+}$ and *Gpihbp1*$^{S/S}$ mice 90 min after the injection of Alexa Fluor 488–11A12. (**C**) Capillary cross sections in BAT 90 min after an injection of Alexa Fluor 488–11A12, 0.75 U heparin, and 15 µg dextran sulfate. (**D**) Capillary cross sections in BAT 90 min after an injection of Alexa Fluor 488–11A12 and 34 µmol of a synthetic peptide corresponding to the wild-type GPIHBP1 AD (EDGDADPEPENYNYDDDDDEEEEEE). (**E**) Capillary cross sections in BAT 90 min after an injection of Alexa Fluor 488–11A12 and 34 µmol of a synthetic peptide corresponding to the S-protein tag ("S-tag") (KETAAAKFERQHMDS).

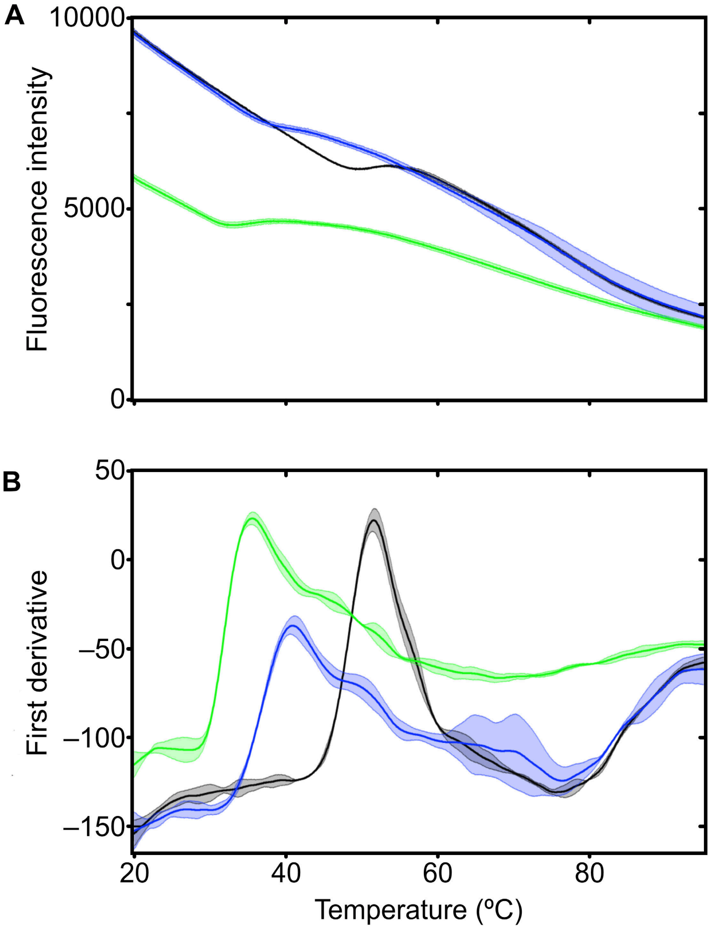

Supplemental figure 10. Thermal stability of purified mouse LPL, alone or when complexed to WT-GPIHBP1 or S-GPIHBP1. The melting temperatures (T_m) for 6 µM mouse LPL alone (*green;* T_m of 34.5 ± 0.5°C); in complex with WT-GPIHBP1 (*black;* T_m of 52.5 ± 0.2°C); or in complex with S-GPIHBP1 (*blue;* T_m of 39.3 ± 0.8°C) were measured by recording fluoresence emission at 330 nm (A) and its first derivative (B) during thermal unfolding with temperature ramping of 1°C per min from 20–95°C. Data are represented as mean ± SD; the shadowed areas of the melting curves depict the SD from three independent experiments.

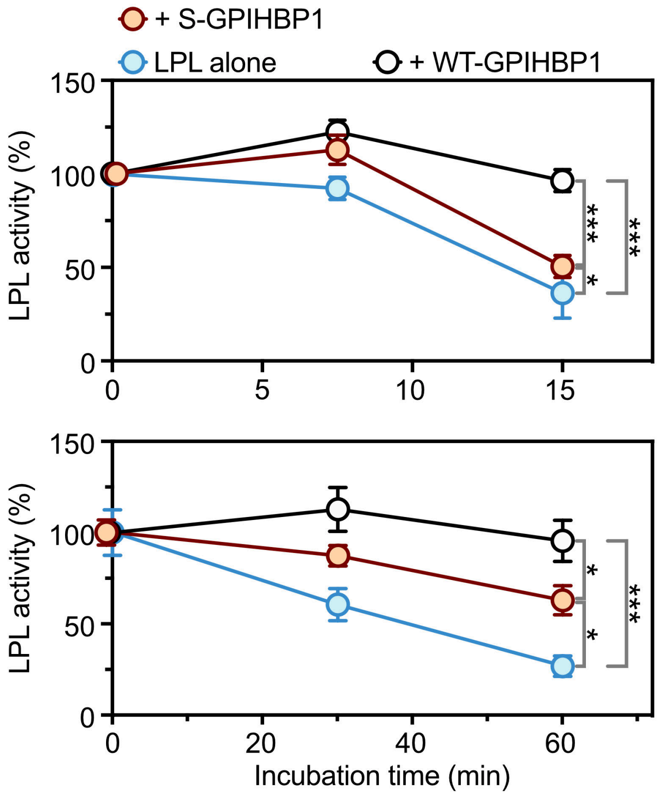

Supplemental figure 11. Stability of the triglyceride hydrolase activity of purified mouse LPL at room temperature, alone or when complexed with WT-GPIHBP1 or S-GPIHBP1. The GPIHBP1:LPL molar ratio was 5:1. Shown are two independent experiments. Data are represented as mean ± SEM (triplicate measurements at each time point). **p* < 0.05; ****p* < 0.001. A two-way ANOVA test was used to compare means.

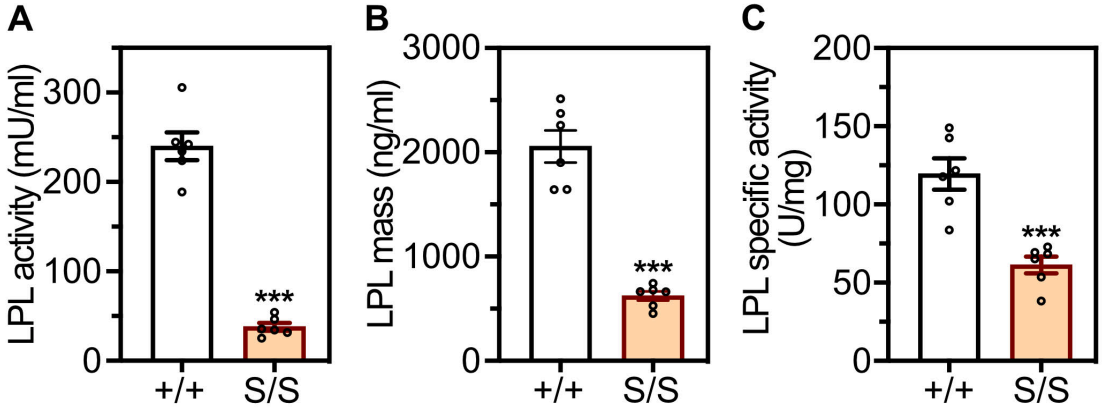

Supplemental figure 12. LPL activity (A), LPL mass (B), and LPL specific activity (C) in the plasma of *Gpihbp1*$^{+/+}$ and *Gpihbp1*$^{S/S}$ mice (n = 6/group) 2 min after an intravenous injection of heparin (15 U). LPL activity was measured with a [^{3}H]triolein substrate; mass was measured with a sandwich ELISA; specific activity was calculated by dividing LPL activity by LPL mass. Data are represented as mean ± SEM. ***$p < 0.001$. A two-tailed Student's t test was used to compare means in panels **A–C**.

Supplemental Table 1. Resource table.

Reagent or Resource	Source	Identifier
**Antibodies**		
Rat monoclonal antibody against GPIHBP1 (11A12)	Beigneux et al., 2009	N/A
Goat polyclonal antibody against CD31	R&D Systems	Cat#AF3628
Hamster monoclonal antibody against CD31 (2H8)	Developmental Studies Hybridoma Bank at the University of Iowa	N/A
Mouse monoclonal antibody against the V5 epitope tag	ThermoFisher Scientific	Cat#R960CUS
Goat polyclonal antibody against the S-protein epitope tag	Abcam	Cat#ab.19321
Goat polyclonal antibody against mouse LPL	Weinstein et al., 2008	N/A
Rat IgG	Vector Laboratories	Cat#I-4000-1
Rabbit polyclonal antibody against β-actin	Novus Biologicals	Cat#NB600-503
IRDye800 donkey anti-goat IgG secondary antibody	LI-COR	Cat#926-32214
IRDye800 donkey anti-rabbit IgG secondary antibody	LI-COR	Cat#926-32213
DyLight650–labeled anti-rat IgG secondary antibody	ThermoFisher Scientific	Cat#SA5-10029
Alexa Fluor 549–labeled anti-hamster IgG secondary antibody	Jackson ImmunoResearch	Cat#307-506-003
Alexa Fluor 488–labeled anti-rabbit IgG secondary antibody	ThermoFisher Scientific	Cat#A21206
Alexa Fluor 568–anti-goat IgG secondary antibody	ThermoFisher Scientific	Cat#A11057
**Bacterial**		
Drosophila melanogaster Schneider 2 (S2) cells	ThermoFisher Scientific	Cat#R69007
**Chemicals, Peptides, and Recombinant proteins**		
[9, 10-^{3}H(N)] triolein	PerkinElmer	Cat#NET43100MC
StartingBlock	ThermoFisher Scientific	Cat#37578
1-Step Ultra TMB-ELISA	ThermoFisher Scientific	Cat#34028
Phosphatidylinositol-specific phospholipase C (PIPLC)	ThermoFisher Scientific	Cat#P6466
ProLong Diamond antifade with DAPI	ThermoFisher Scientific	Cat#P36962
OCT compound	Fisher HealthCare	Cat#4585

Heparin sulfate	McKesson Corporation	NDC. 63739-931-14
Dextran sulfate	Calbiochem	Cat#3730-OP
GPIHBP1 acidic domain peptide	Genescript	Custom made
Peptide corresponding to the S-protein epitope tag	Genescript	Custom made
CellFectin II Reagent	ThermoFisher Scientific	Cat#10362100
Schneider's *Drosophila* medium (SDM)	ThermoFisher Scientific	Cat#21720024
Express-Five serum free medium (SFM)	ThermoFisher Scientific	Cat#10486025
Hygromycin B	ThermoFisher Scientific	Cat#10687010
12% Bis-Tris SDS-PAGE	NuPAGE, ThermoFisher Scientific	Cat#NP0342BOX
Recombinant mouse LPL	In-house production in *Drosophila* S2 cells	N/A
**Critical Commercial Assays**		
Serum Triglyceride Determination Kit	Millipore Sigma	Cat#TR0100
Cell line T nucleofector kit	Lonza	Cat#VCA-1002
Alexa Fluor 488 Protein Labeling Kit	ThermoFisher Scientific	Cat#A10235
Alexa Fluor 555 Protein Labeling Kit	ThermoFisher Scientific	Cat#A20174
Alexa Fluor 647 Protein Labeling Kit	ThermoFisher Scientific	Cat#A20173
DyLight 680 Antibody Labeling Kit	ThermoFisher Scientific	Cat#53056
DyLight 800 Antibody Labeling Kit	ThermoFisher Scientific	Cat#53062
**Recombinant DNA**		
pTriEx4-Stag-mouse GPIHBP1 plasmid (wt or mutant), Novagen [mammalian (CMV) expression vector backbone]	Beigneux et al., 2007; Beigneux et al., 2011	
pcDNA6-mouse LPL-V5/His plasmid, ThermoFisher scientific [mammalian (CMV) expression vector backbone]	Allan et al., 2016	
**Software and Algorithms**		
Prism v9.0.2	Graphpad	https://www.graphpad.com/
Waters MassLynx Mass Spectrometry Software	Waters Corporation	https://www.waters.com/

Image J v2.1.0/1.53c	NIH	https://imagej.nih.gov/ij/
ImageStudioLite v5.2.5	LI-COR	https://www.licor.com/bio/image-studio-lite/
Affinity Designer v1.9.3	Serif Labs	https://affinity.serif.com/en-us/designer/
Affinity Photo v1.9.3	Serif Labs	https://affinity.serif.com/en-us/photo/
Zen blue v3.3	Zeiss	https://www.zeiss.com/microscopy/int/products/microscope-software/zen.html
T200 Evaluation Software v3.0	GE Healthcare	https://www.cytivalifesciences.com/en/us/shop/protein-analysis/spr-label-free-analysis/software/
PR.ThermControl	Nanotemper	https://nanotempertech.com/prometheus-pr-thermcontrol-software/
PR.Analysis	Nanotemper	https://nanotempertech.com/prometheus/
**Other**		
Heparin–Sepharose HiTrap columns (1 ml)	GE Healthcare	Cat#17040601
Synapt G2 Tri-Wave Ion Mobility Mass Spectrometer	Waters Corporation	https://www.waters.com/
SpectraMax iD3 plate reader	Molecular Devices	
LSM980 microscope	Zeiss	
Infrared scanner	LI-COR	
Biacore T200	GE Healthcare	
Äkta Purifier	GE Healthcare	
Prometheus NT. 48	Nanotemper	https://nanotempertech.com/prometheus/

Supplemental Table 2. qPCR primer sequence.

Gene	Forward	Reverse
Gpihbp1	AGCAGGGACAGAGCACCTCT	AGACGAGCGTGATGCAGAAG
Lpl	AGGTGGACATCGGAGAACTG	TCCCTAGCACAGAAGATGACC
Cd31	AACCGTATCTCCAAAGCCAGT	CCAGACGACTGGAGGAGAACT
Cd36	GGCCAAGCTATTGCGACAT	CAGATCCGAACACAGCGTAGA

Full unedited gel for Figure 1F

Upper panel

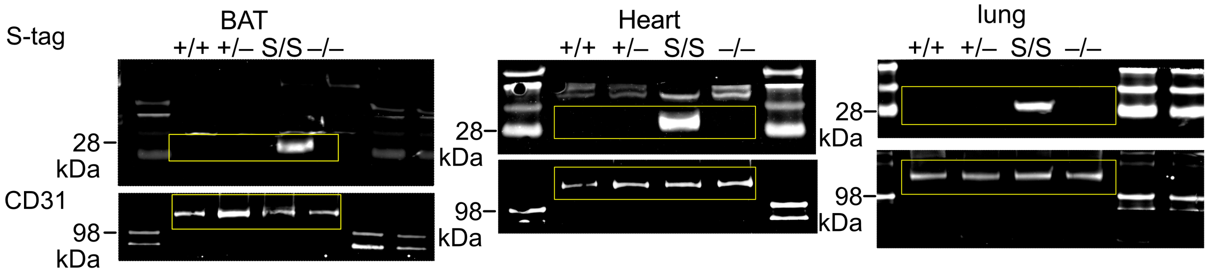

Bottom panel

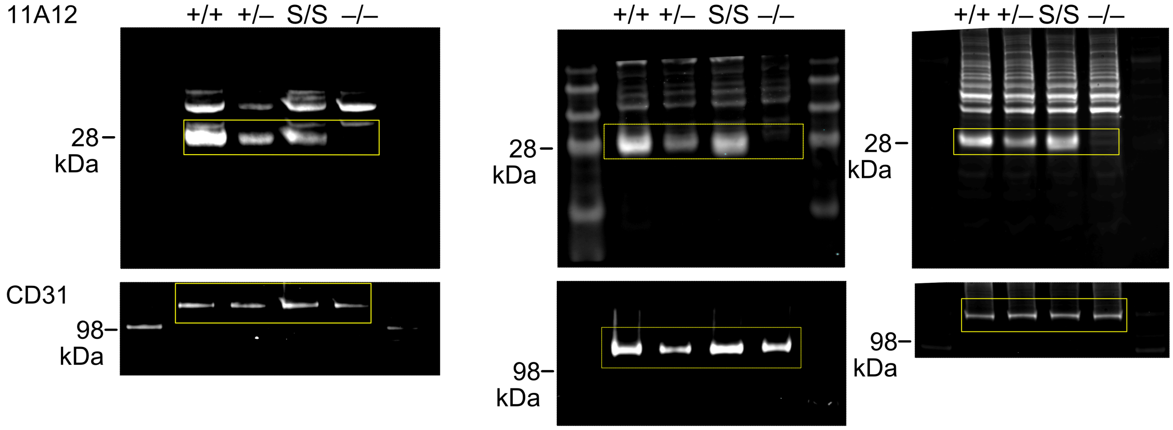

Full unedited gel for Figure 3C

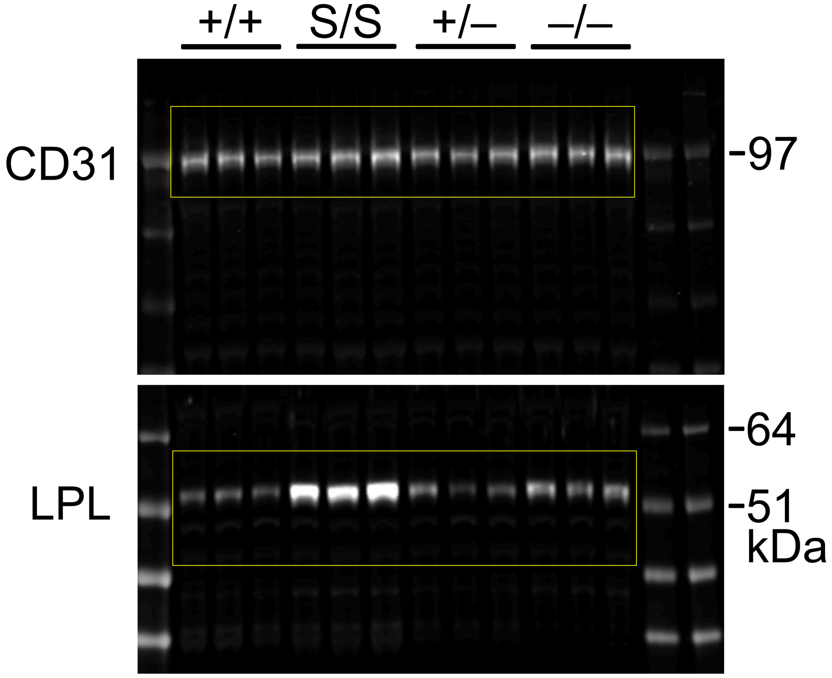

Full unedited gel for Figure 6B

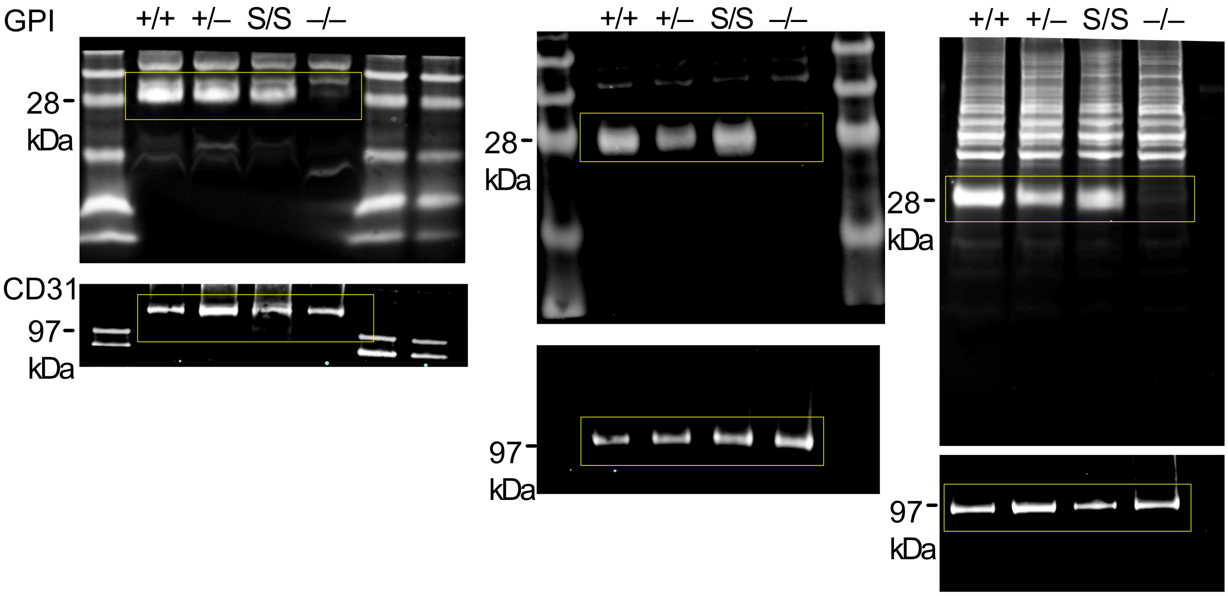

Full unedited gel for Supplemental figure 1F

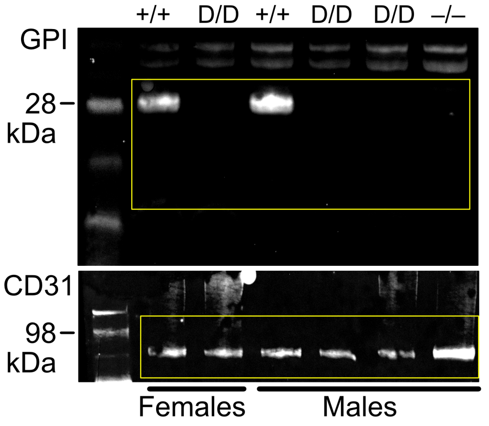

Full unedited gel for Supplemental figure 2D

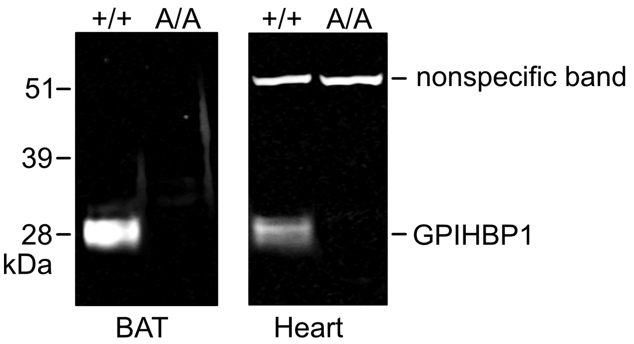

Full unedited gel for Supplemental figure 3A

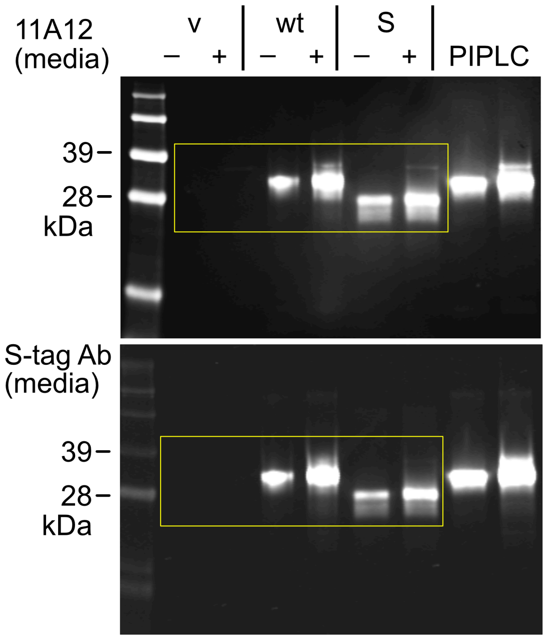

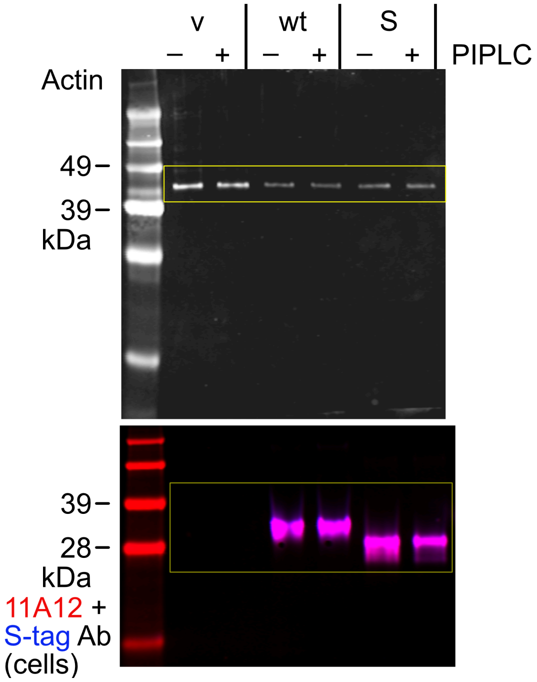

Full unedited gel for Supplemental figure 7C

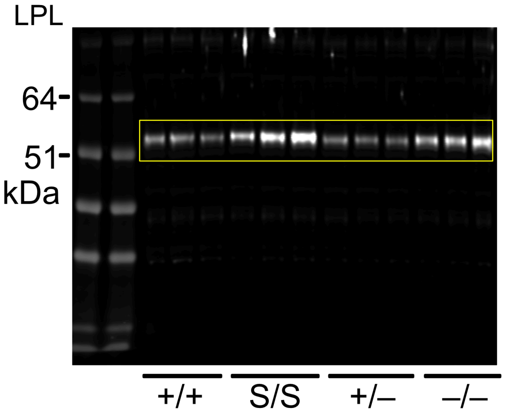

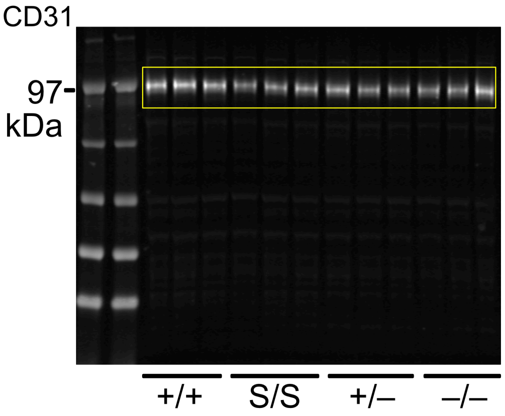

Supplement: Supplemental data [file jci-132-157500-s037.pdf]
